# Supplementary material for: A Concise Diastereoselective Total Synthesis of α-Ambrinol
Source: Mar Drugs. 2023 Apr 1;21(4):230. doi: 10.3390/md21040230 (PMC10145093; doi:10.3390/md21040230)
Supplement: Supplementary file 1 [file marinedrugs-21-00230-s001.zip › marinedrugs-2306126-supplementary.pdf]

Supporting Information

A concise diastereoselective total synthesis of α-ambrinol

Josefa L. López-Martínez[1], Irene Torres-García[1], Irene Moreno-Gutiérrez[1], Pascual Oña Burgos[2], Antonio Rosales-Martínez[3], Manuel Muñoz-Dorado[1], Miriam Álvarez-Corral[1]*, Ignacio Rodríguez-García[1]*.

1 Organic Chemistry, University of Almería, CIAIMBITAL. E04120 Almería, Spain; pepaloma91@hotmail.com (J.L.L.-M.); irene.tg.94@gmail.com (I.T.-G.); irenemorenogtrz@gmail.com (I.M.-G.); mdorado@ual.es (M.M.-D.); malvarez@ual.es (M.A.-C.); irodrigu@ual.es (I.R.-G.)

2 Instituto de Tecnología Química, Universitat Politècnica de València-Consejo Superior de Investigaciones Científicas (UPV-CSIC), 46022 Valencia, Spain; pasoabur@itq.upv.es

3 Department of Chemical Engineering, Escuela Politécnica Superior, University of Sevilla, 41011 Sevilla, Spain; arosales@us.es

* Correspondence: irodrigu@ual.es.

NMR AND IR SPECTRA

^{1}H NMR, DEPT 135, ^{13}C NMR and IR of **12**........1

^{1}H NMR, DEPT 135, ^{13}C NMR, HSQC, HMBC, COSY and IR of **cis-21**........4

^{1}H NMR, DEPT 135, ^{13}C NMR, HSQC, HMBC, COSY, NOESY 1D and IR of **23**........11

^{1}H NMR, DEPT 135, ^{13}C NMR, HSQC and IR of **20**........18

^{1}H NMR, DEPT 135, ^{13}C NMR, HSQC, HMBC, COSY, NOESY 1D and IR of **6**........22

^{1}H NMR, DEPT 135, ^{13}C NMR, HSQC, HMBC, COSY, NOESY 1D and IR of **24**........30

^{1}H NMR of **25**........38

^{1}H NMR, DEPT 135, ^{13}C NMR and IR of **22**........39

^{1}H NMR, DEPT 135, ^{13}C NMR, HSQC, HMBC, COSY and IR of **26**........42

^{1}H NMR, DEPT 135, ^{13}C NMR and IR of 12

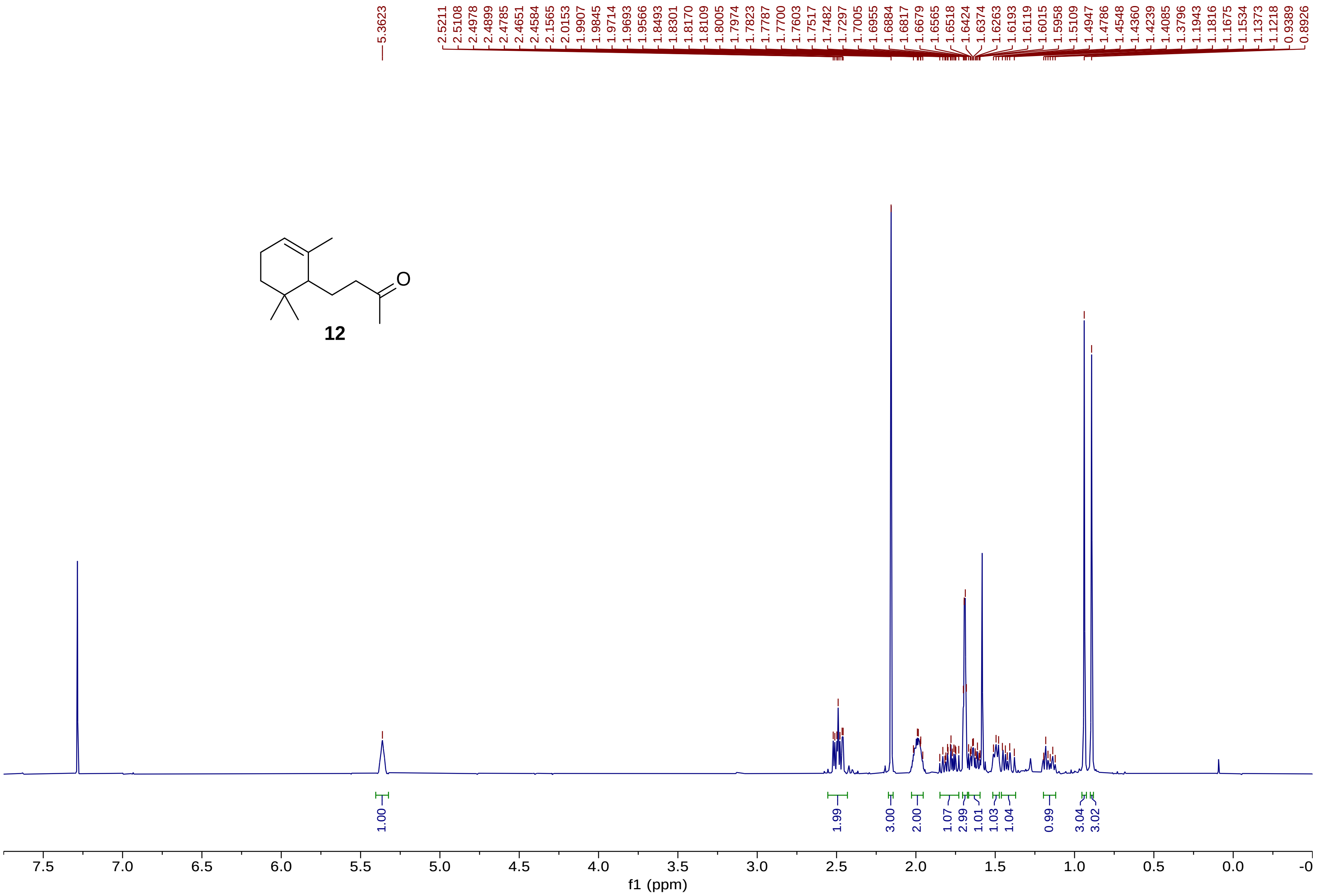

Figure S1

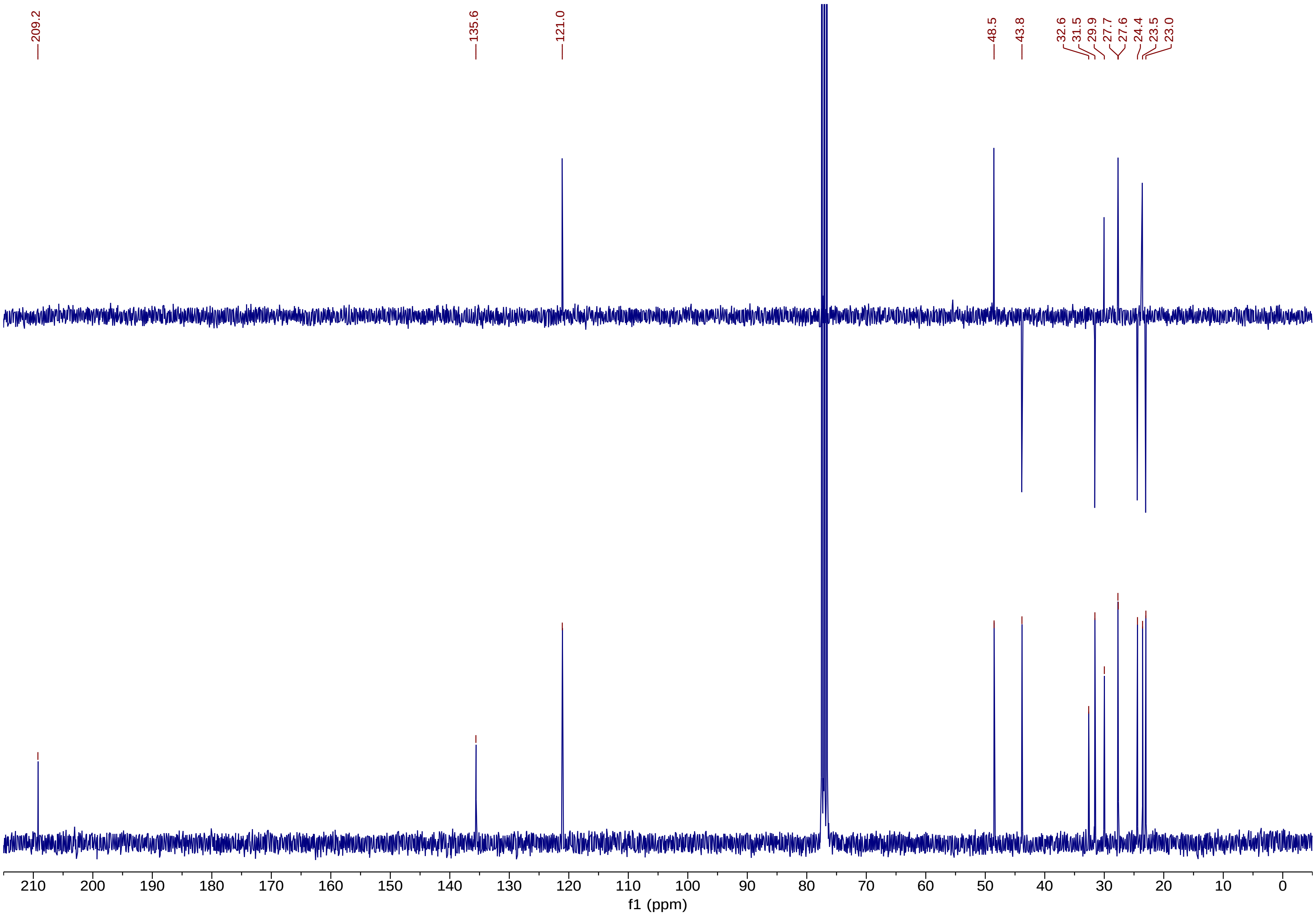

209.2
135.6
121.0
48.5
43.8
32.6
31.5
29.9
27.7
27.6
24.4
23.5
23.0
210 200 190 180 170 160 150 140 130 120 110 100 90 80 70 60 50 40 30 20 10 0
f1 (ppm)

Figure S2

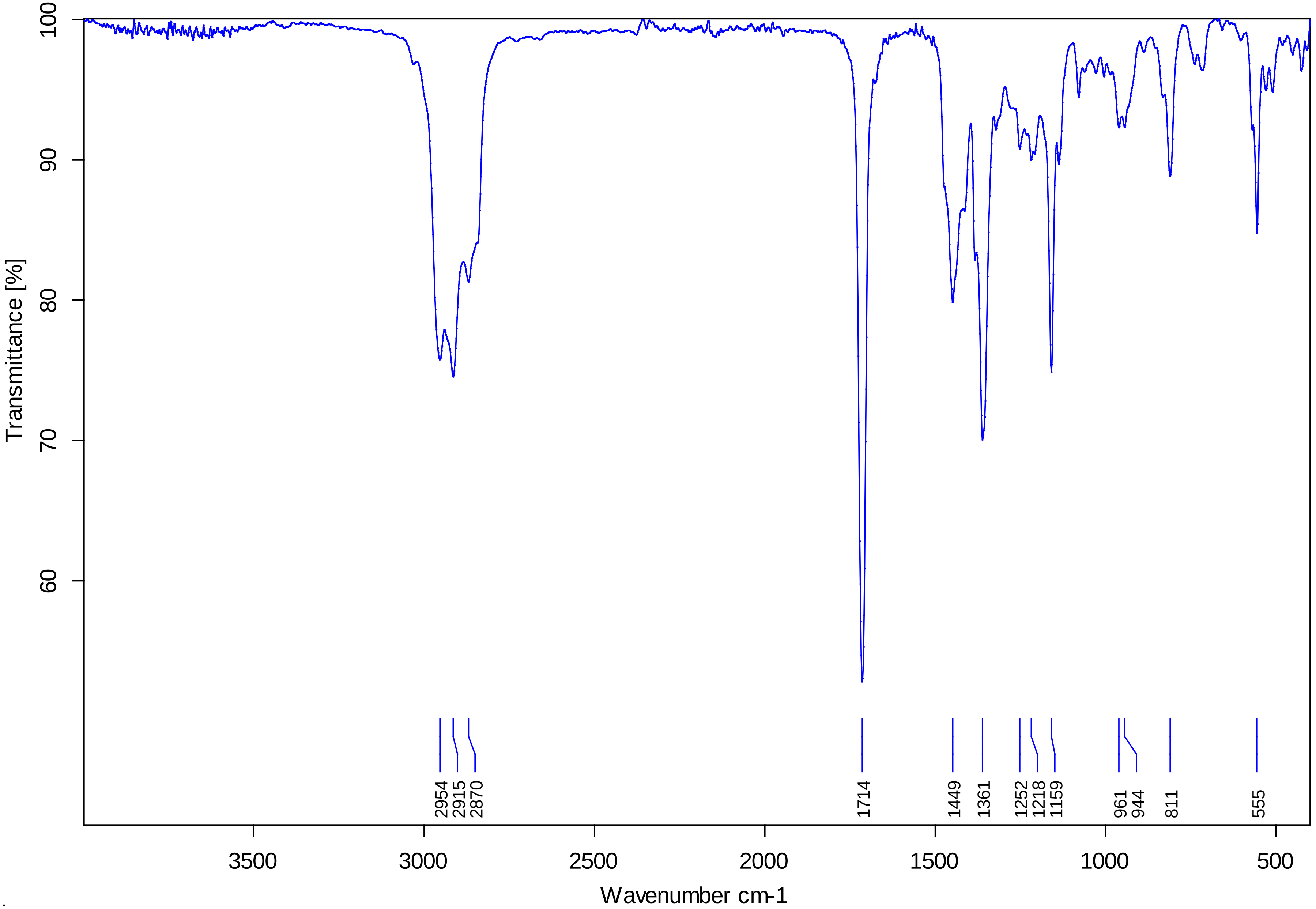
Transmittance [%]
100
90
80
70
60
2954
2915
2870
1714
1449
1361
1252
1218
1159
961
944
811
555
3500
3000
2500
2000
1500
1000
500
Wavenumber cm-1

Figure S3

^{1}H NMR, DEPT 135, ^{13}C NMR, HSQC, HMBC, COSY and IR of *cis*-21

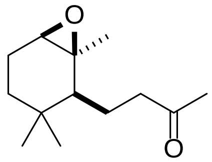

***cis*-21**

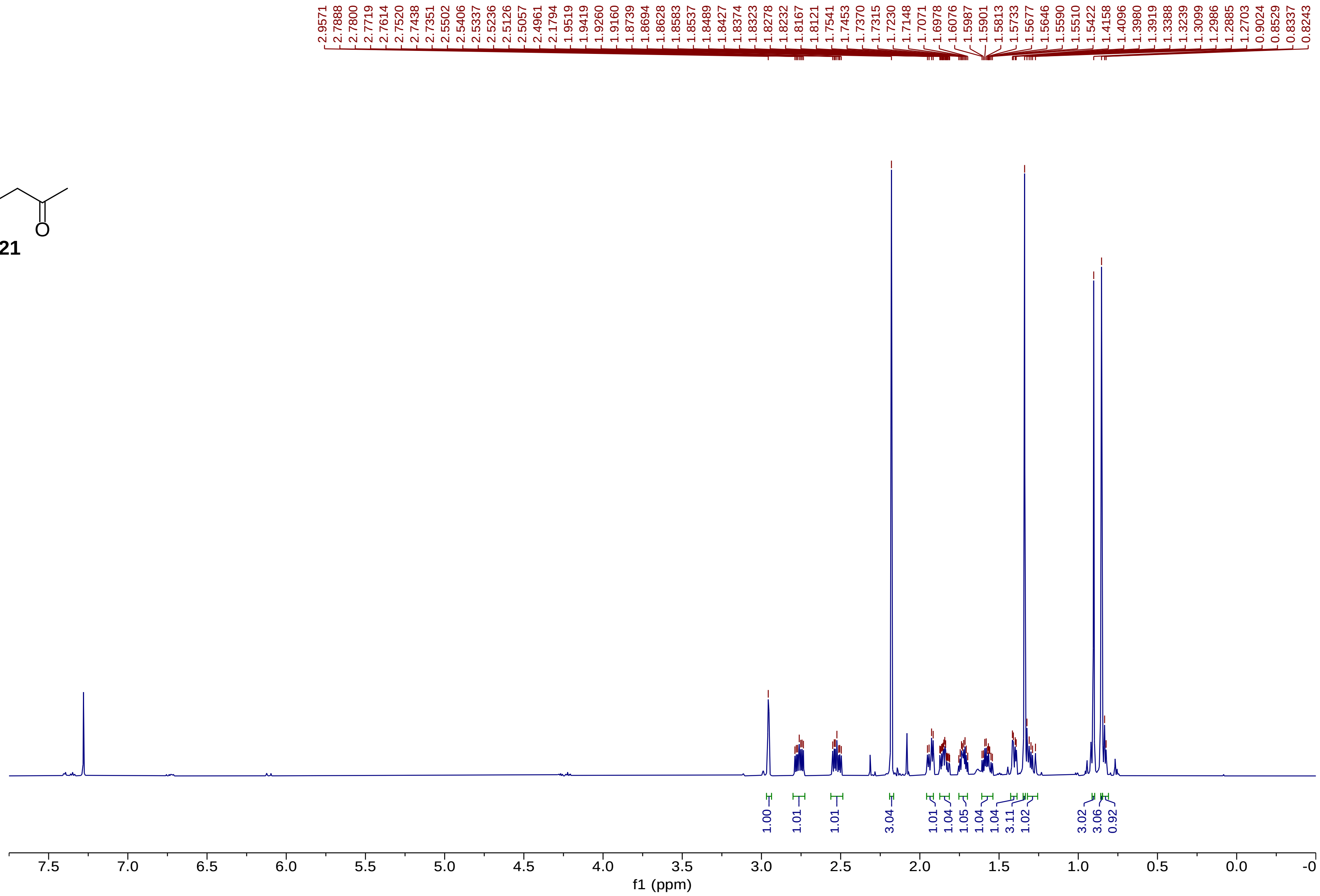

Figure S4

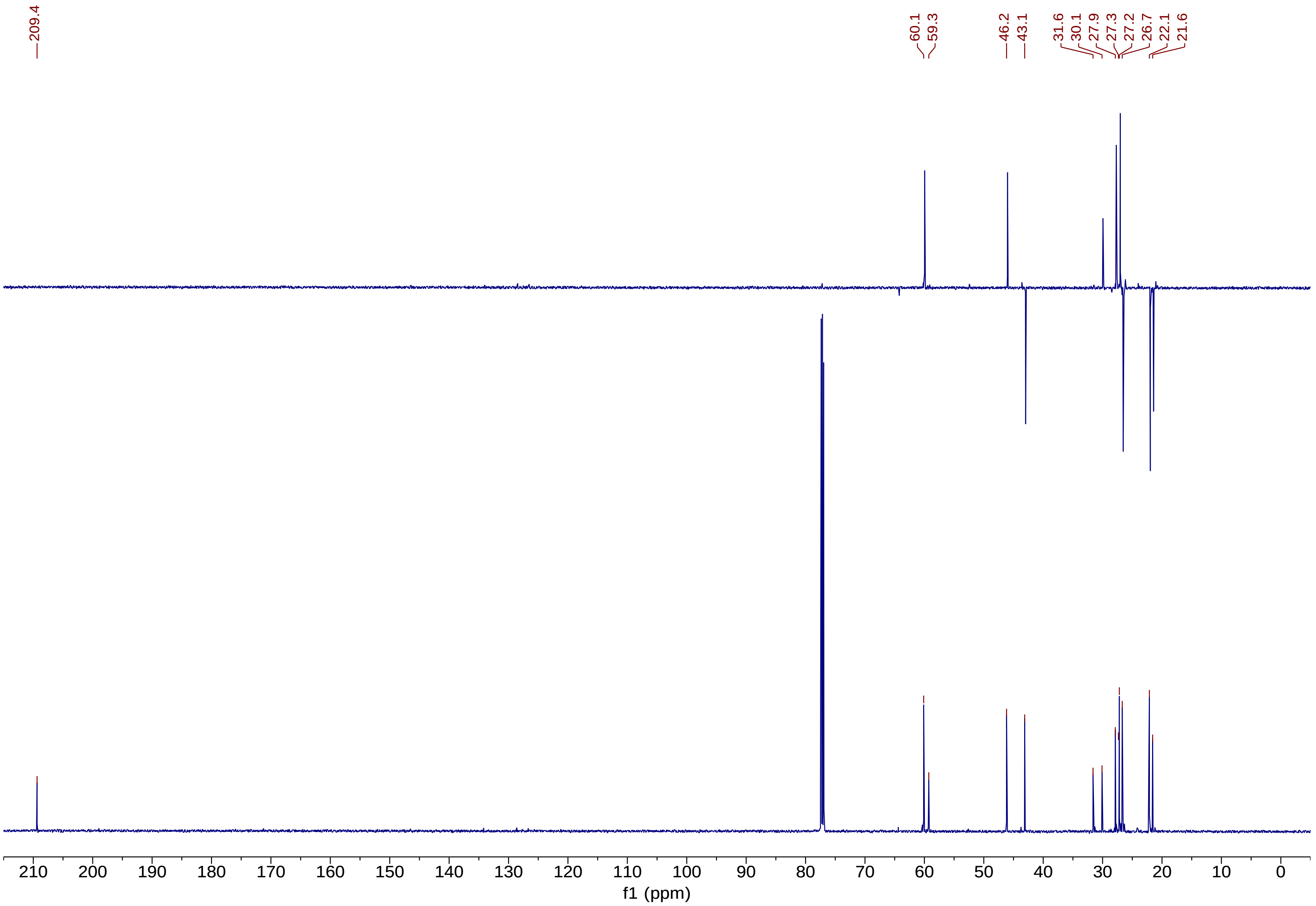

209.4
60.1
59.3
46.2
43.1
31.6
30.1
27.9
27.3
27.2
26.7
22.1
21.6
210
200
190
180
170
160
150
140
130
120
110
100
90
80
70
60
50
40
30
20
10
0
f1 (ppm)

Figure S5

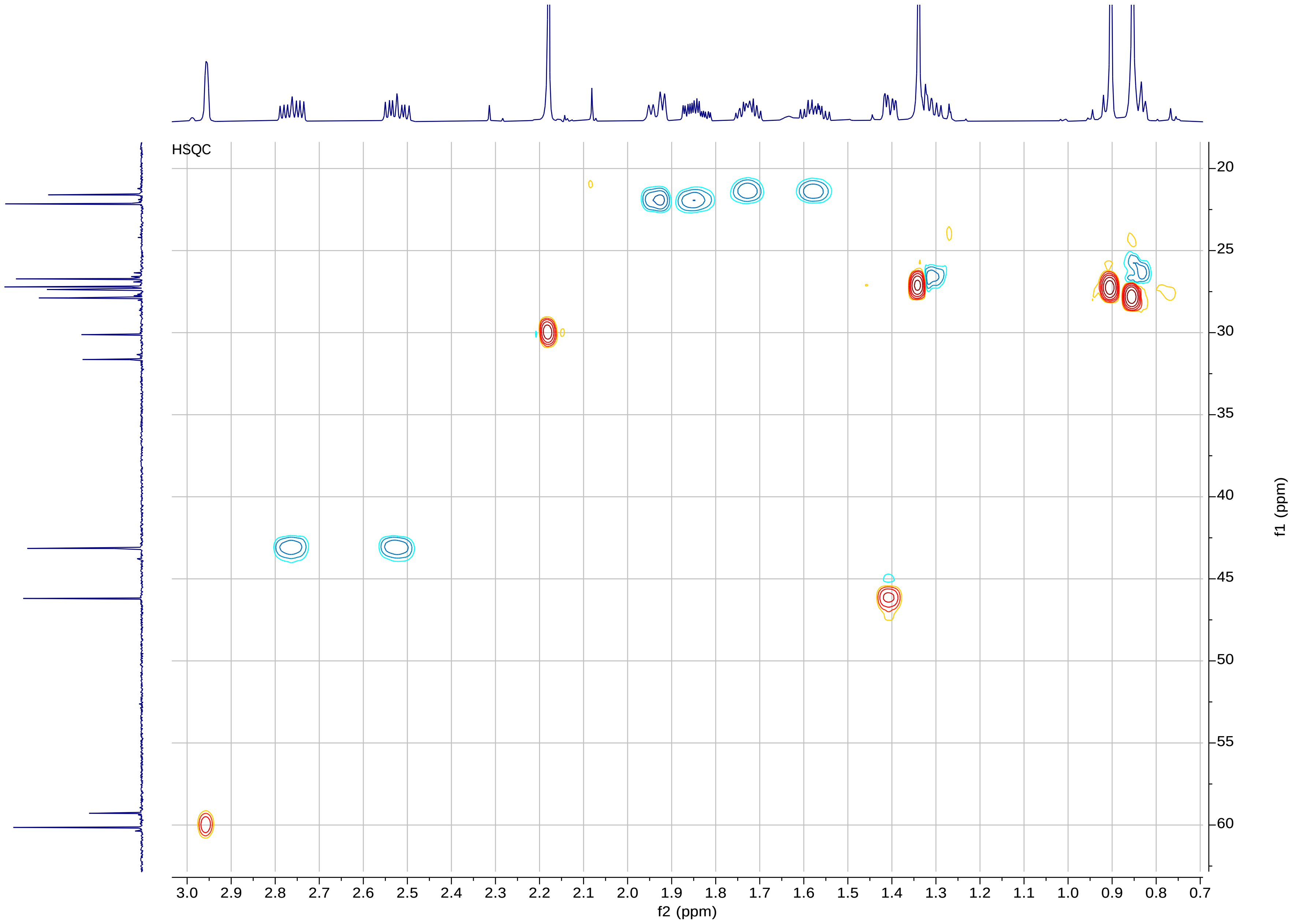

Figure S6

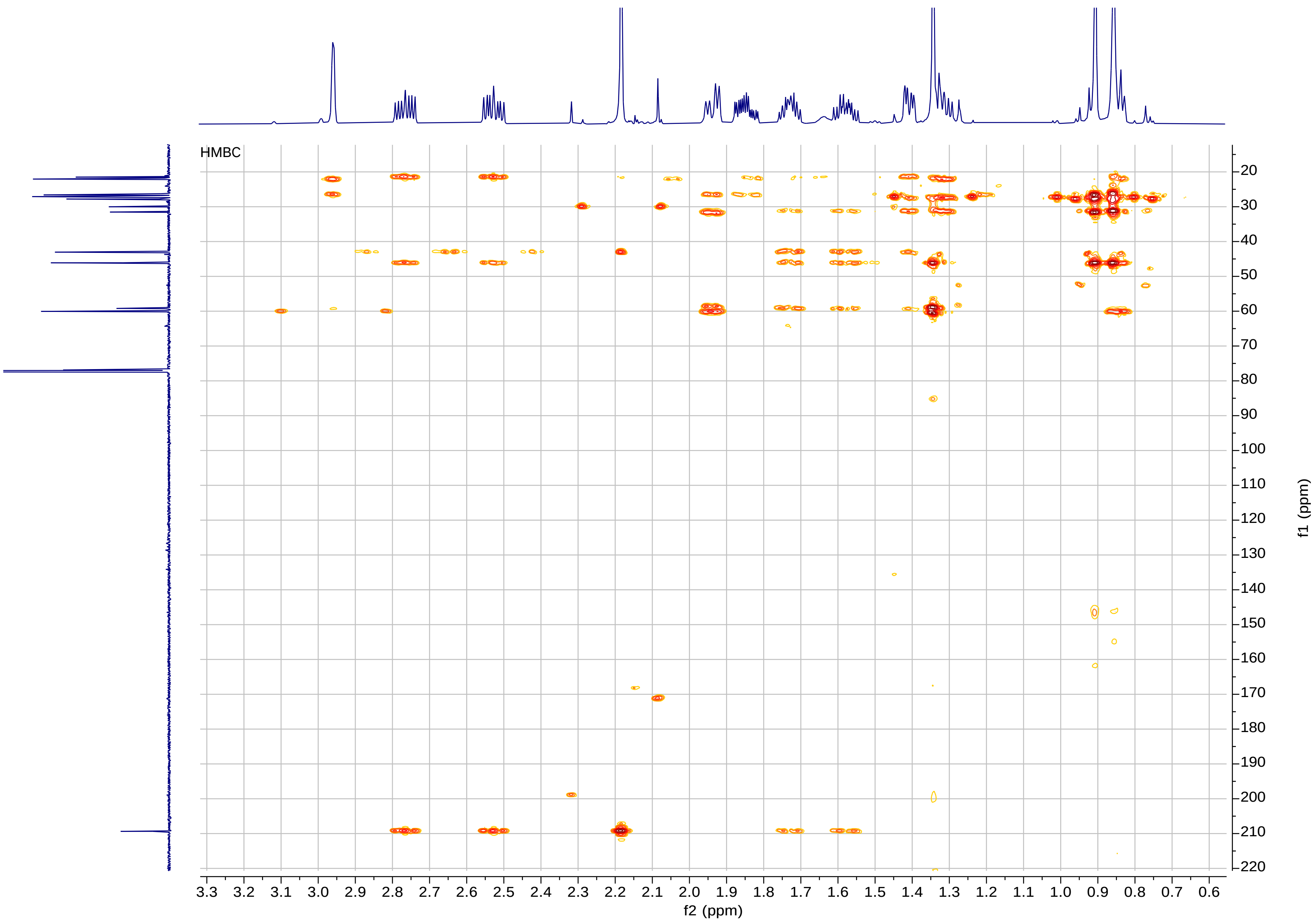
HMBC
3.3
3.2
3.1
3.0
2.9
2.8
2.7
2.6
2.5
2.4
2.3
2.2
2.1
2.0
1.9
1.8
1.7
1.6
1.5
1.4
1.3
1.2
1.1
1.0
0.9
0.8
0.7
0.6
f2 (ppm)
20
30
40
50
60
70
80
90
100
110
120
130
140
150
160
170
180
190
200
210
220
f1 (ppm)

Figure S7

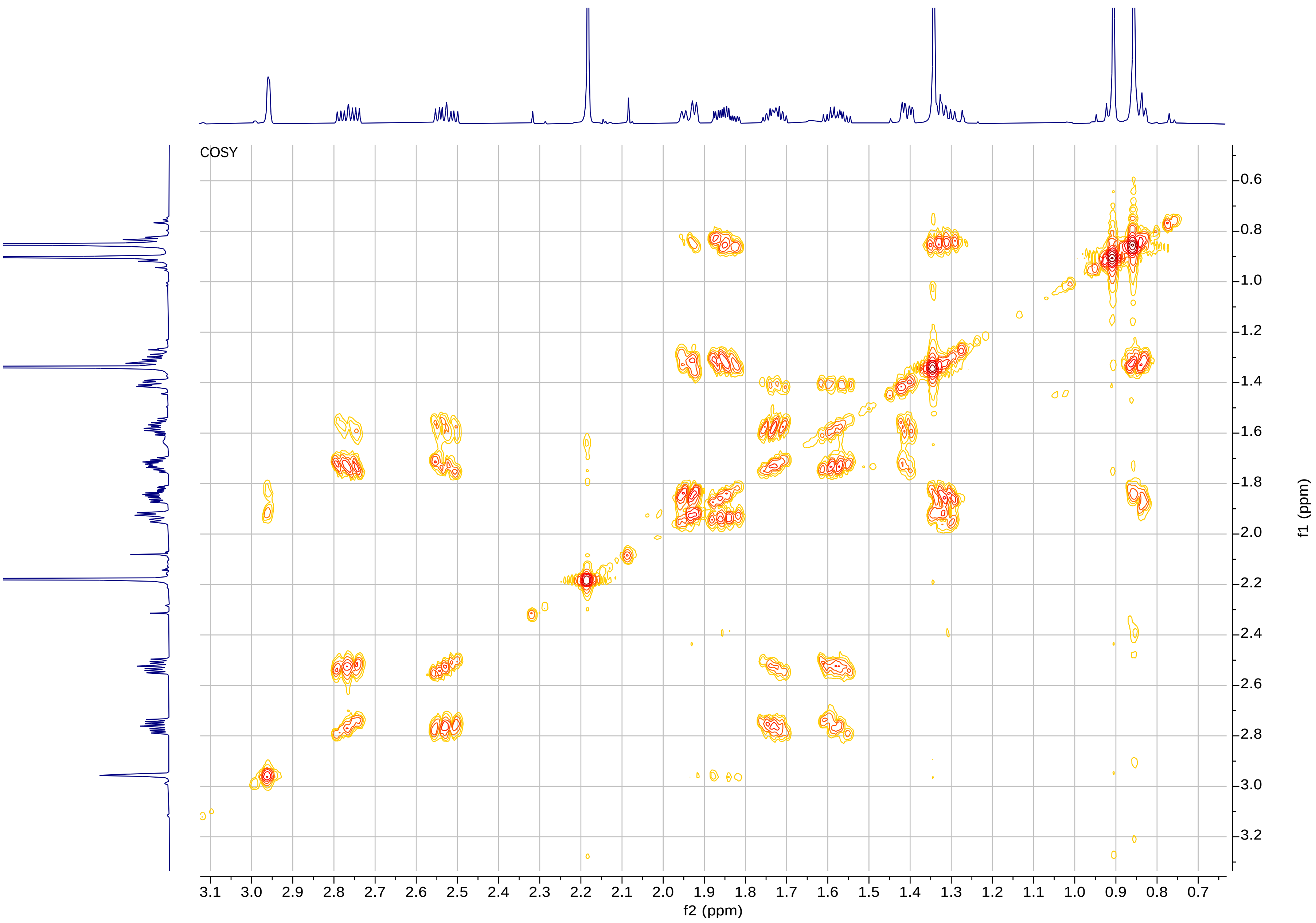

Figure S8

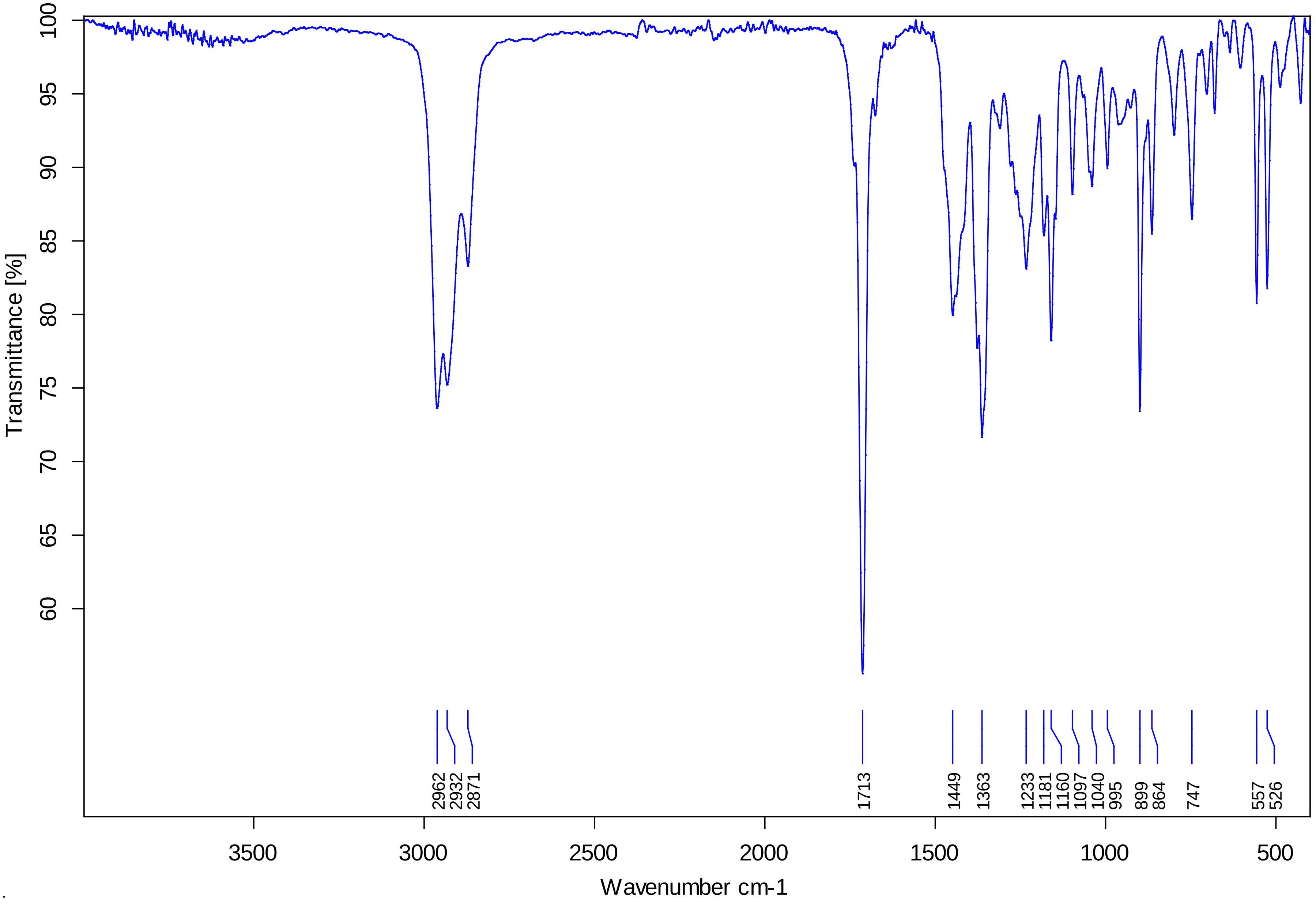
100
95
90
85
80
75
70
65
60
Transmittance [%]
2962
2932
2871
1713
1449
1363
1233
1181
1160
1097
1040
995
899
864
747
557
526
3500
3000
2500
2000
1500
1000
500
Wavenumber cm-1

Figure S9

^{1}H NMR of a 6.67:1 mixture of ***cis*-21** and ***trans*-21** (reaction crude ratio)

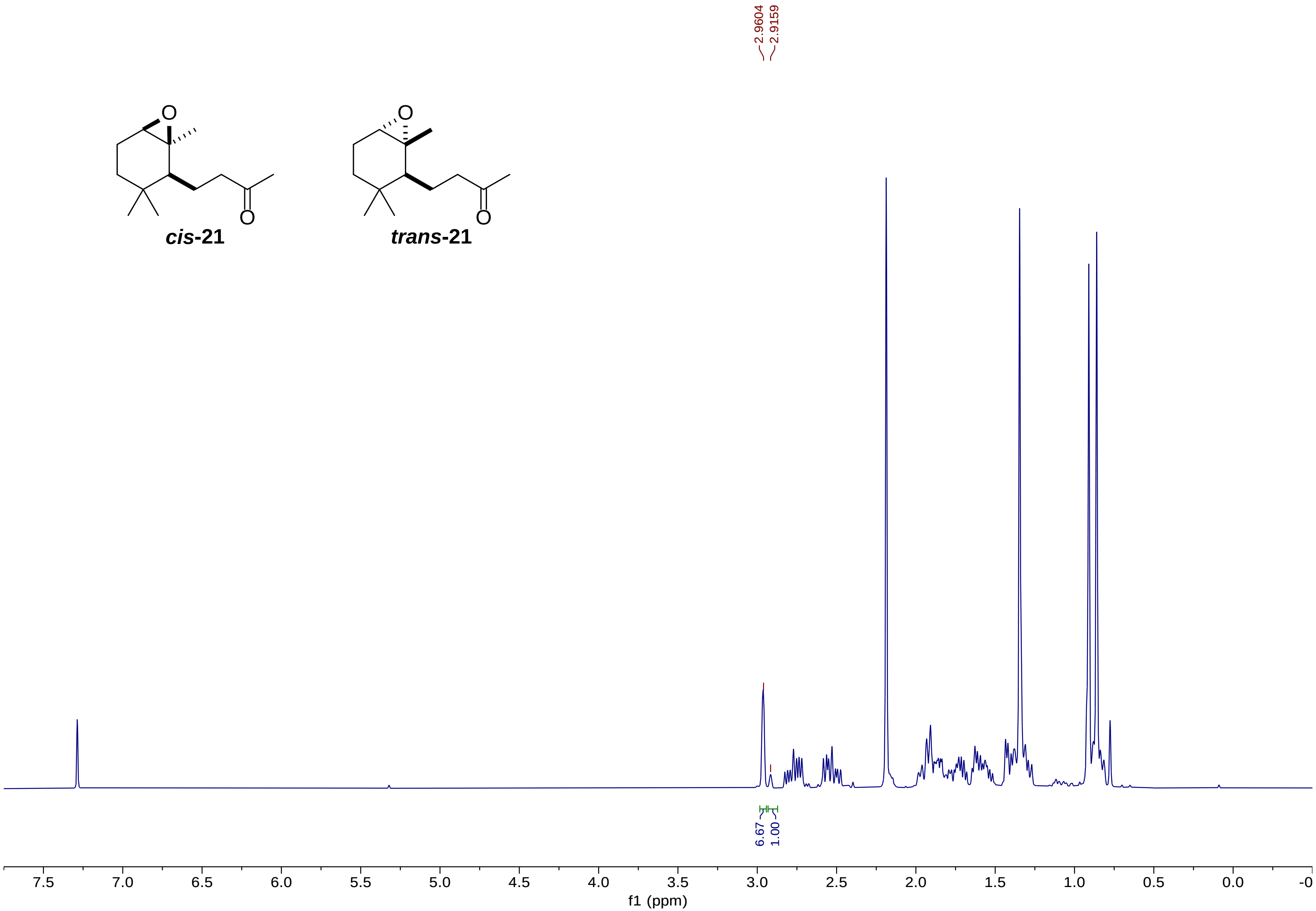

Figure S10

^{1}H NMR, DEPT 135, ^{13}C NMR, HSQC, HMBC, COSY, NOESY 1D and IR of 23

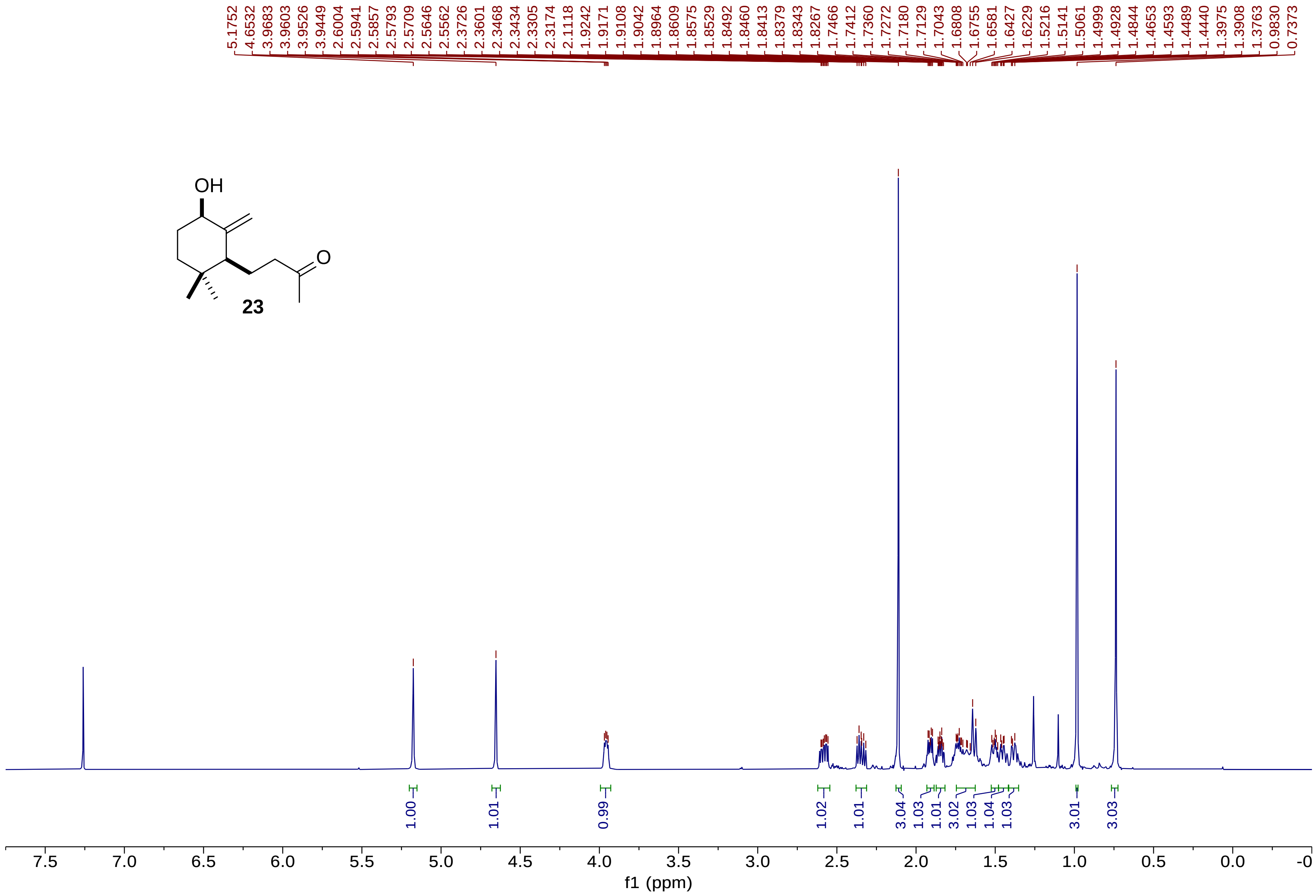

Figure S11

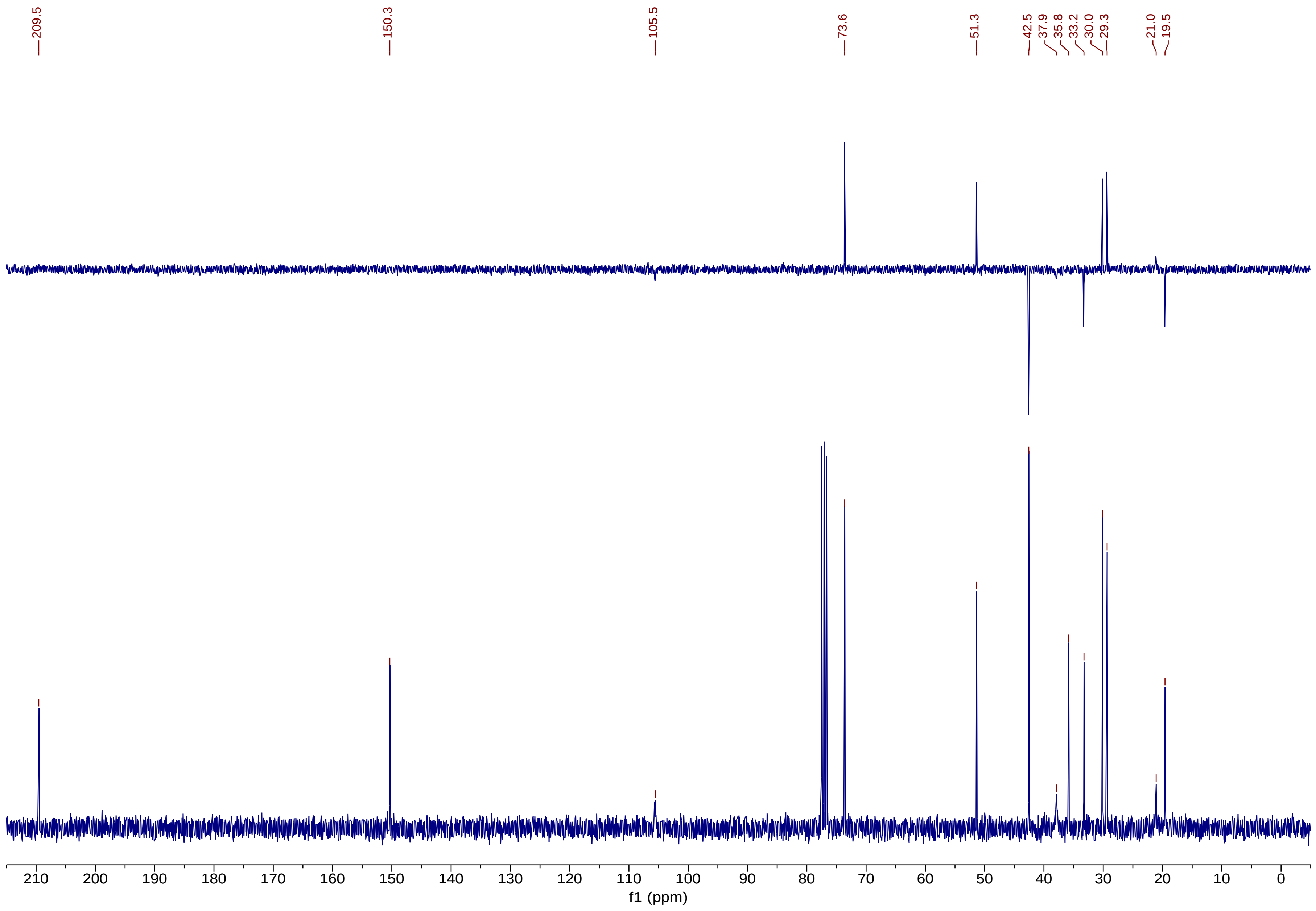
209.5
150.3
105.5
73.6
51.3
42.5
37.9
35.8
33.2
30.0
29.3
21.0
19.5
210 200 190 180 170 160 150 140 130 120 110 100 90 80 70 60 50 40 30 20 10 0
f1 (ppm)

Figure S12

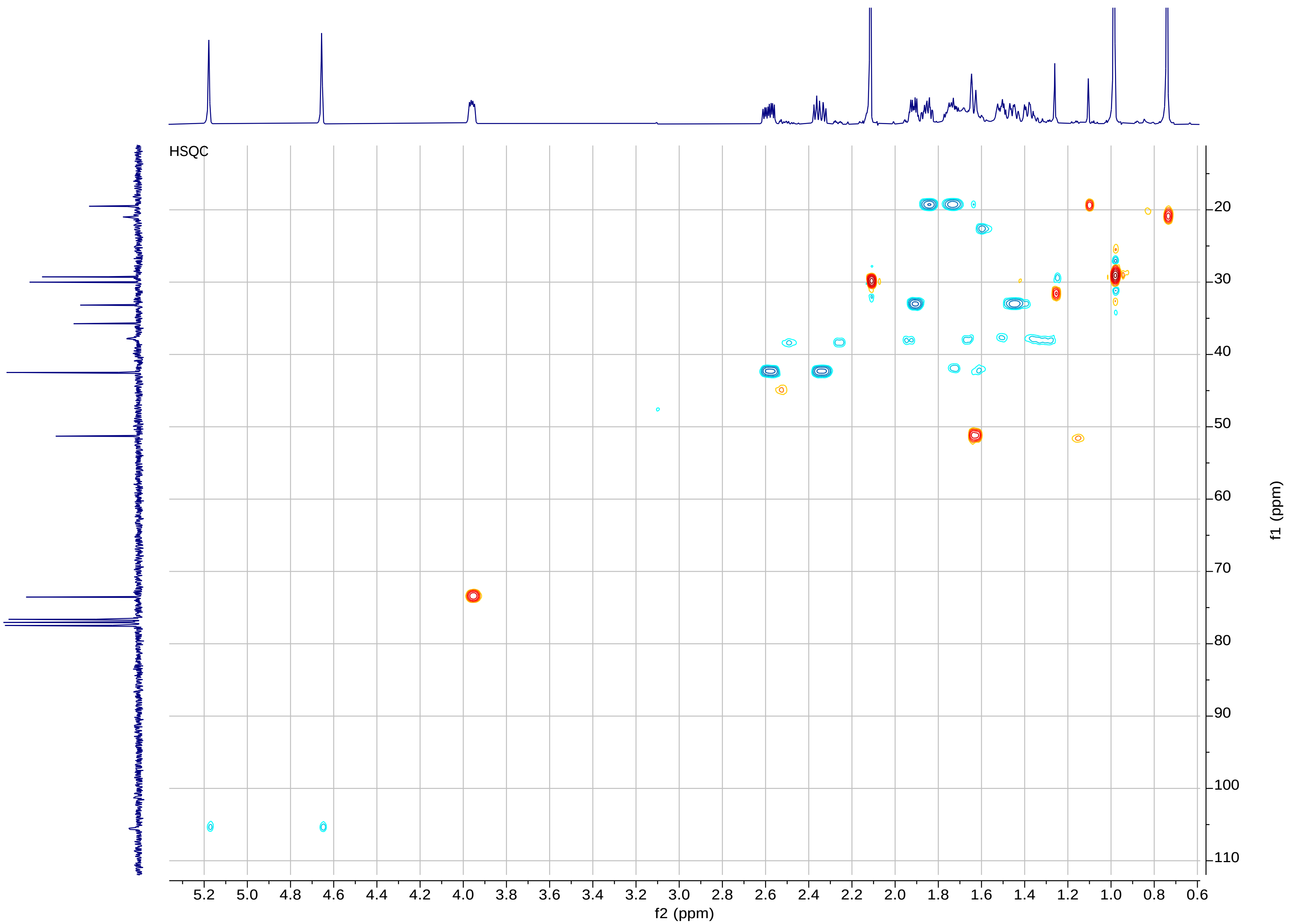

Figure S13

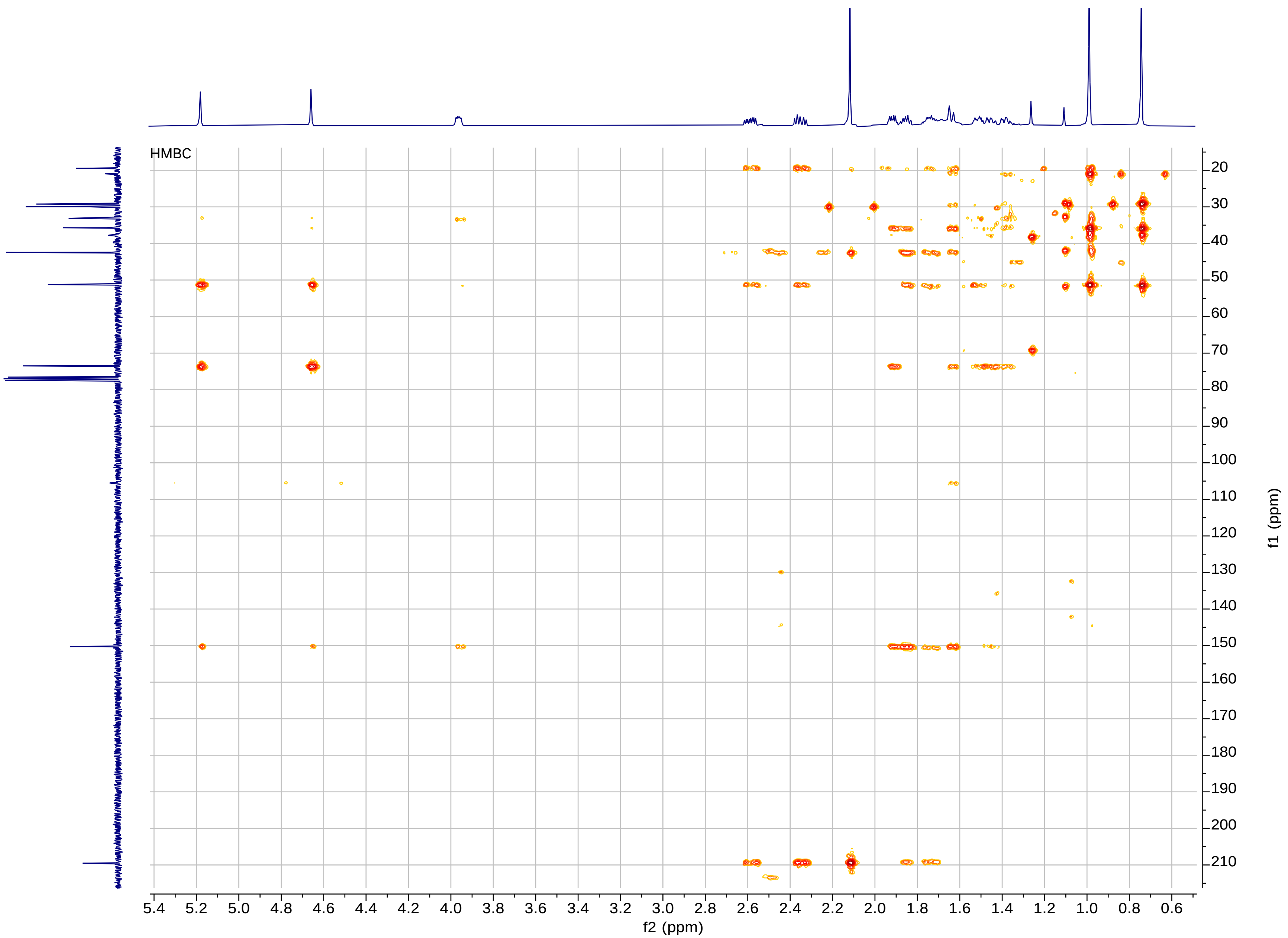

Figure S14

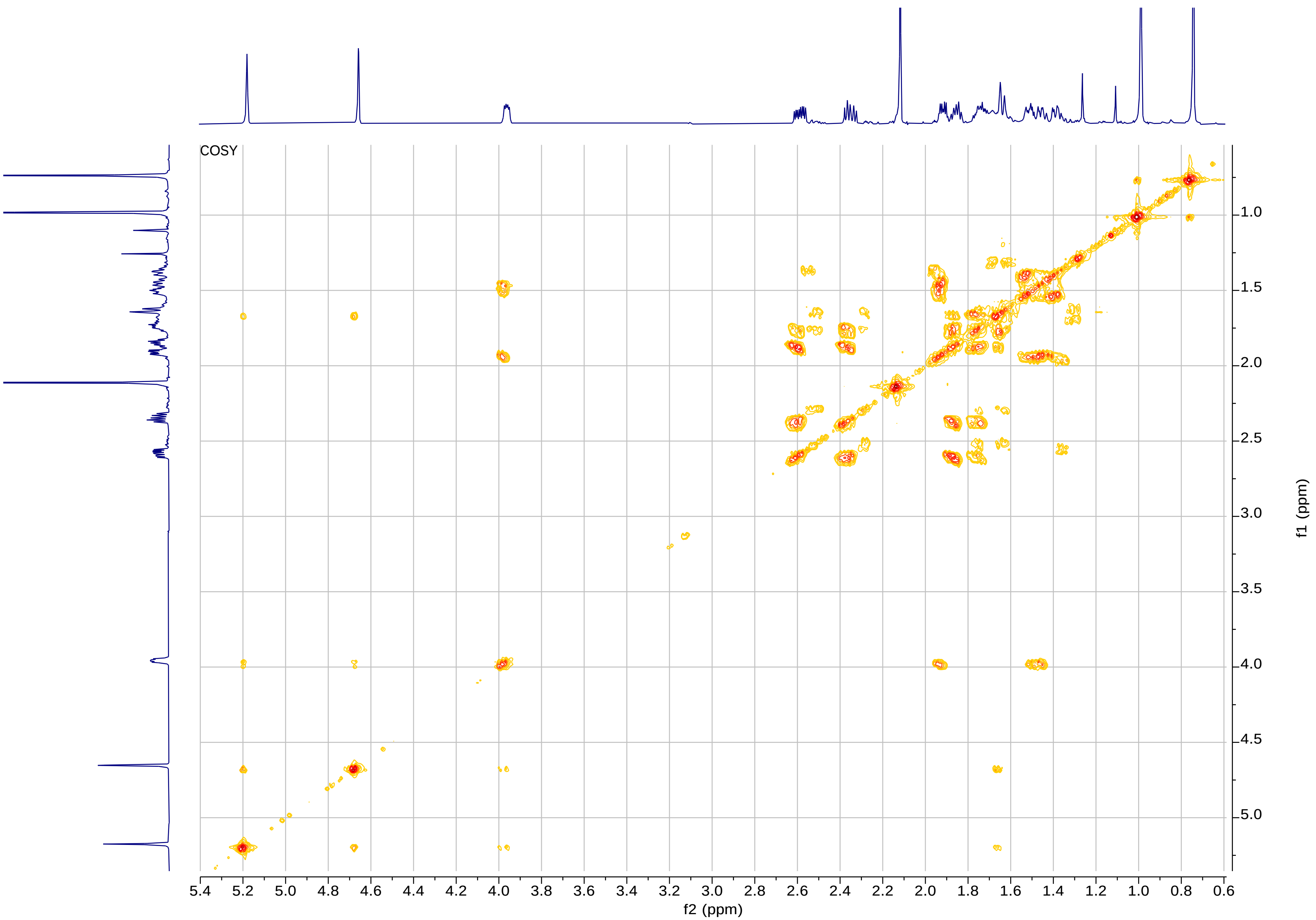
COSY
f2 (ppm)
f1 (ppm)
5.4
5.2
5.0
4.8
4.6
4.4
4.2
4.0
3.8
3.6
3.4
3.2
3.0
2.8
2.6
2.4
2.2
2.0
1.8
1.6
1.4
1.2
1.0
0.8
0.6
1.0
1.5
2.0
2.5
3.0
3.5
4.0
4.5
5.0

Figure S15

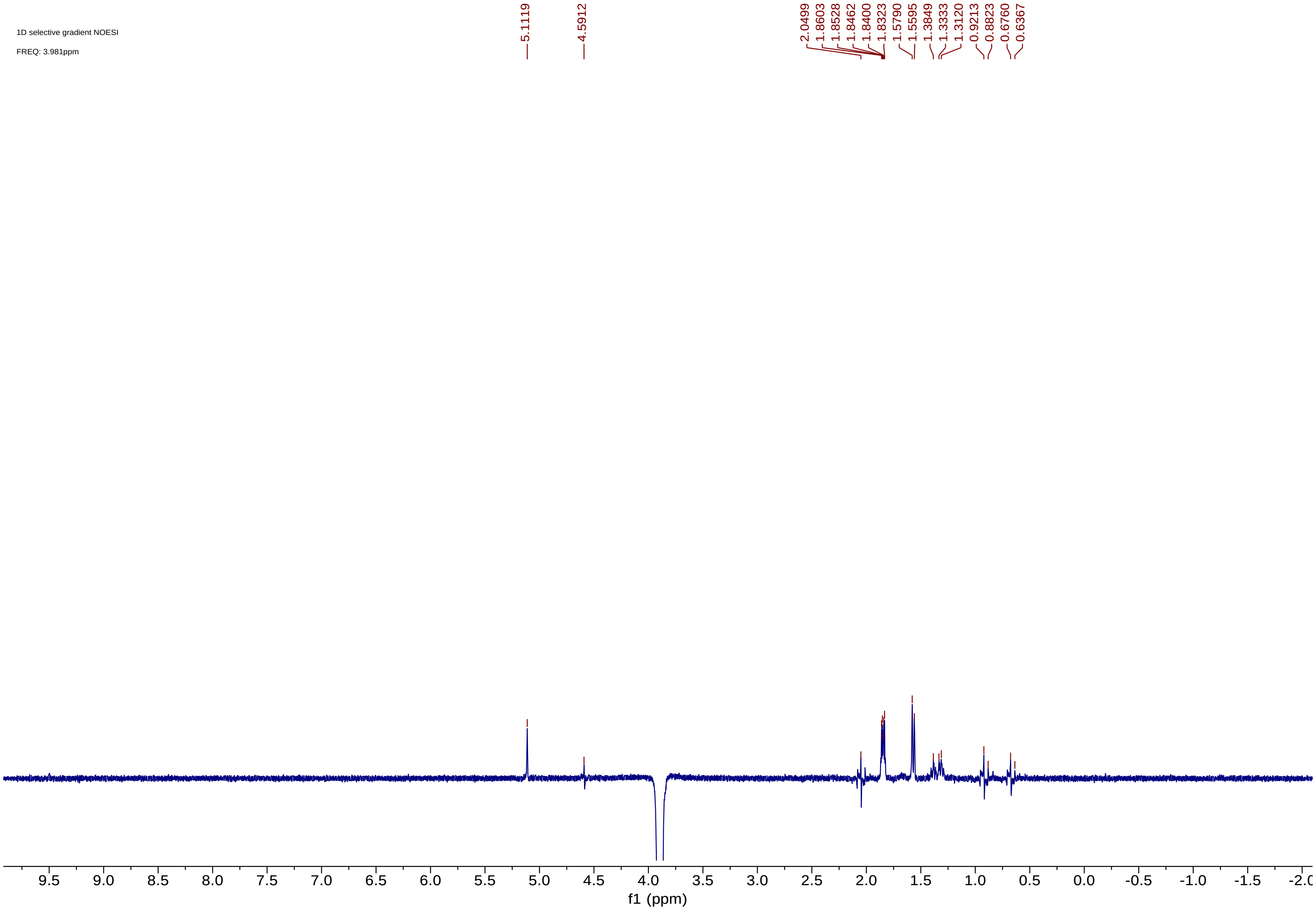
1D selective gradient NOESI
FREQ: 3.981ppm
5.1119
4.5912
2.0499
1.8603
1.8528
1.8462
1.8400
1.8323
1.5790
1.5595
1.3849
1.3333
1.3120
0.9213
0.8823
0.6760
0.6367
f1 (ppm)

Figure S16

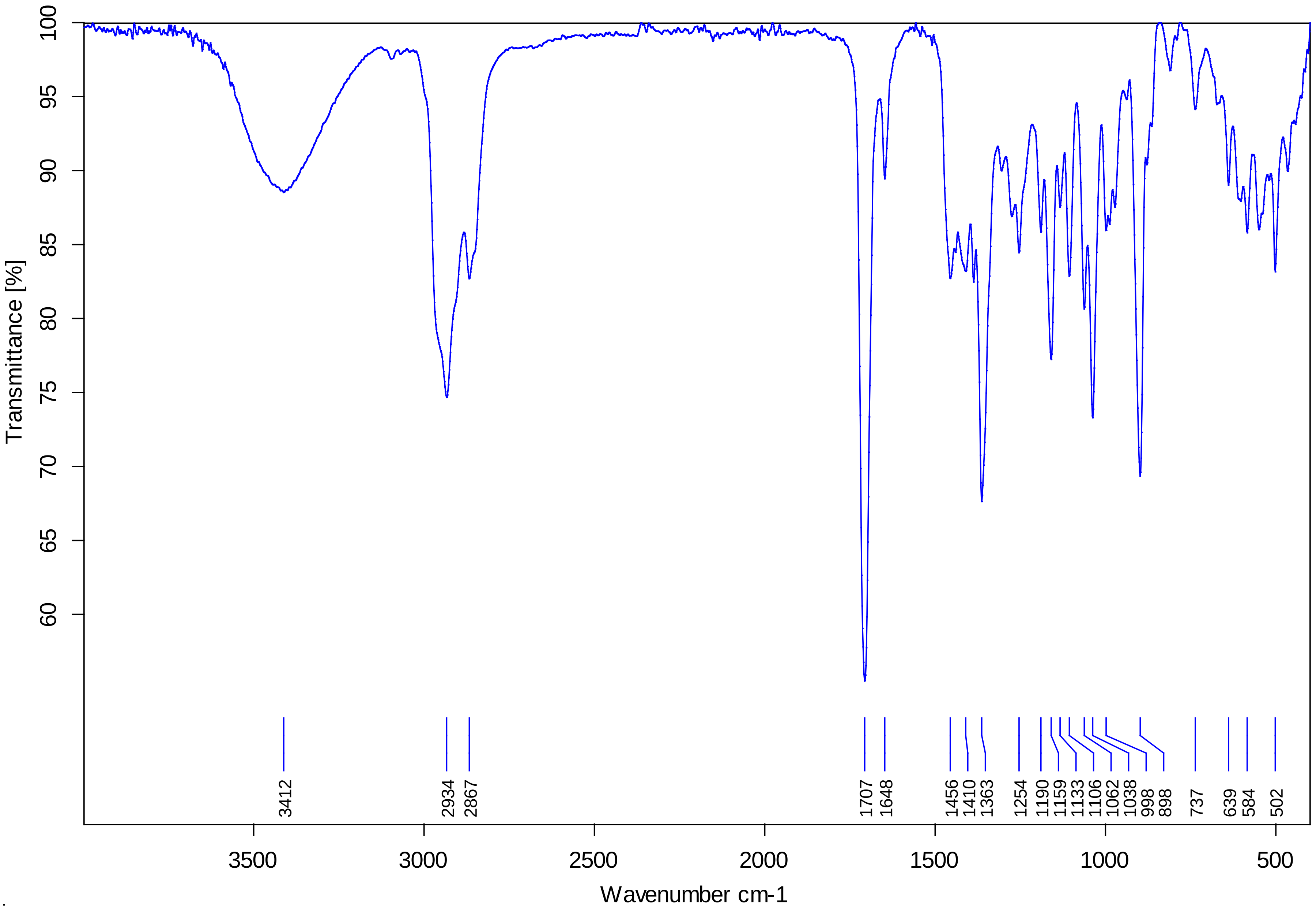
Transmittance [%]
100
95
90
85
80
75
70
65
60
3412
2934
2867
1707
1648
1456
1410
1363
1254
1190
1159
1133
1106
1062
1038
998
898
737
639
584
502
3500
3000
2500
2000
1500
1000
500
Wavenumber cm-1

Figure S17

^{1}H NMR, DEPT 135, ^{13}C NMR, HSQC and IR of 20

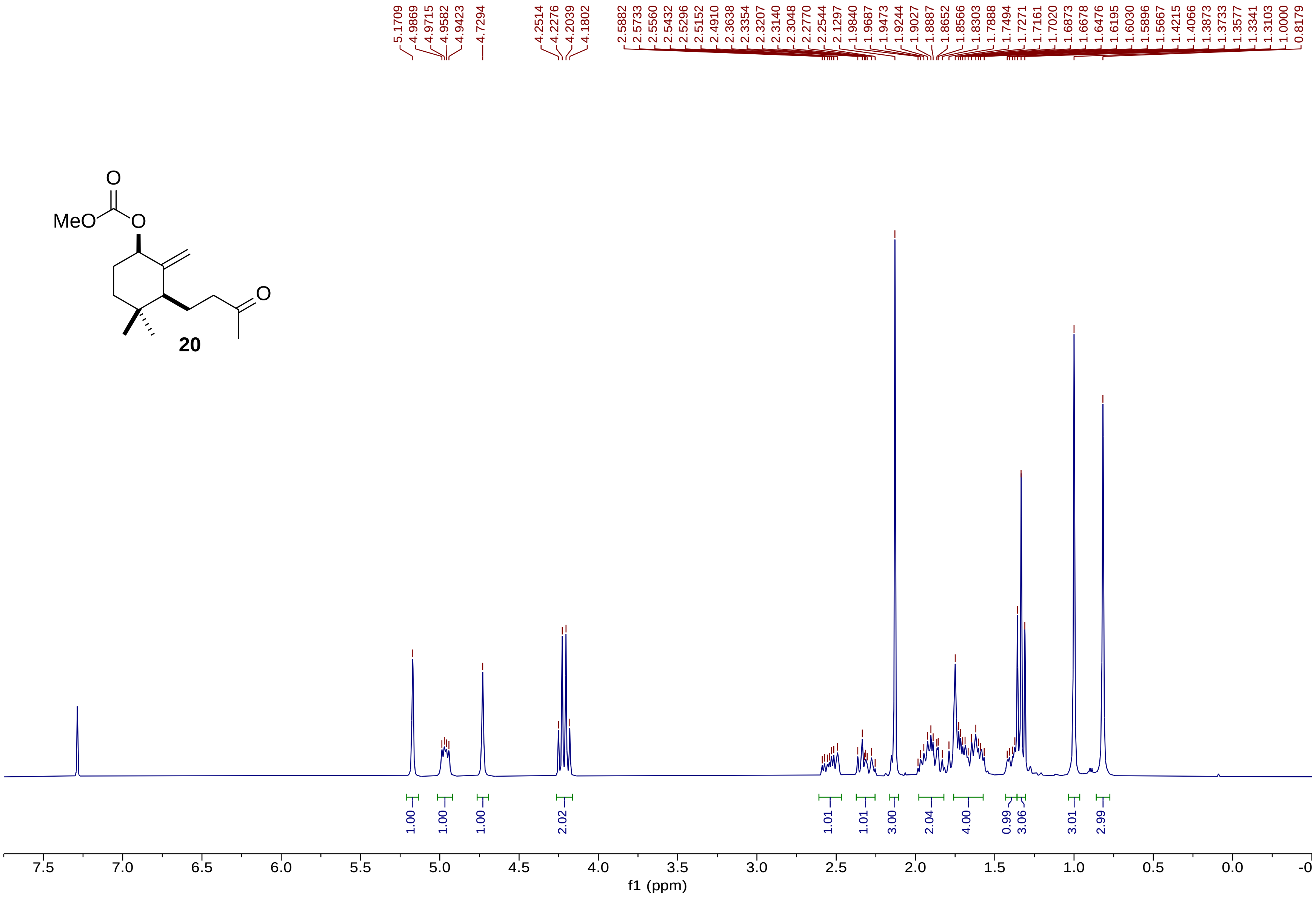

Figure S18

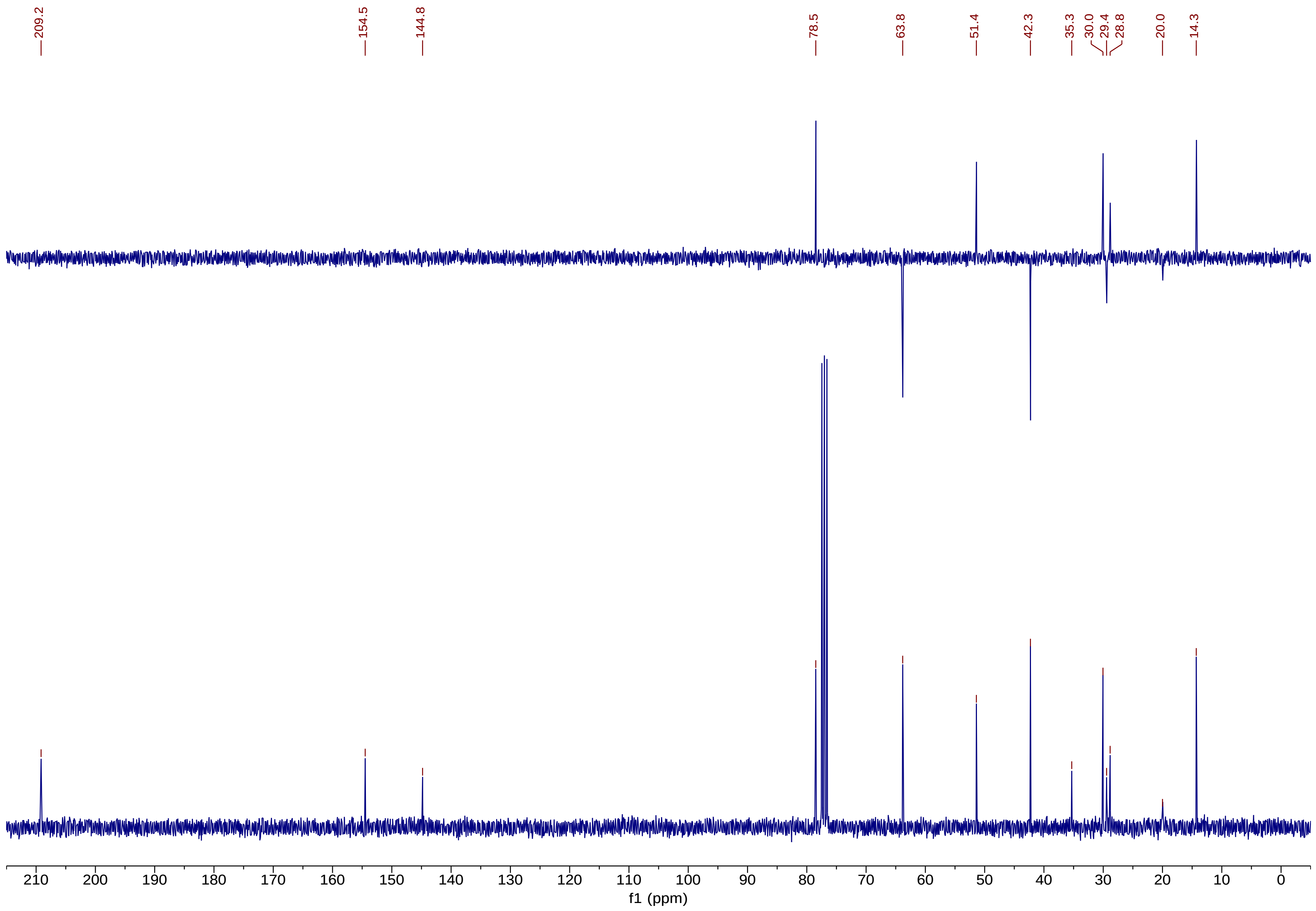
209.2
154.5
144.8
78.5
63.8
51.4
42.3
35.3
30.0
29.4
28.8
20.0
14.3
210
200
190
180
170
160
150
140
130
120
110
100
90
80
70
60
50
40
30
20
10
0
f1 (ppm)

Figure S19

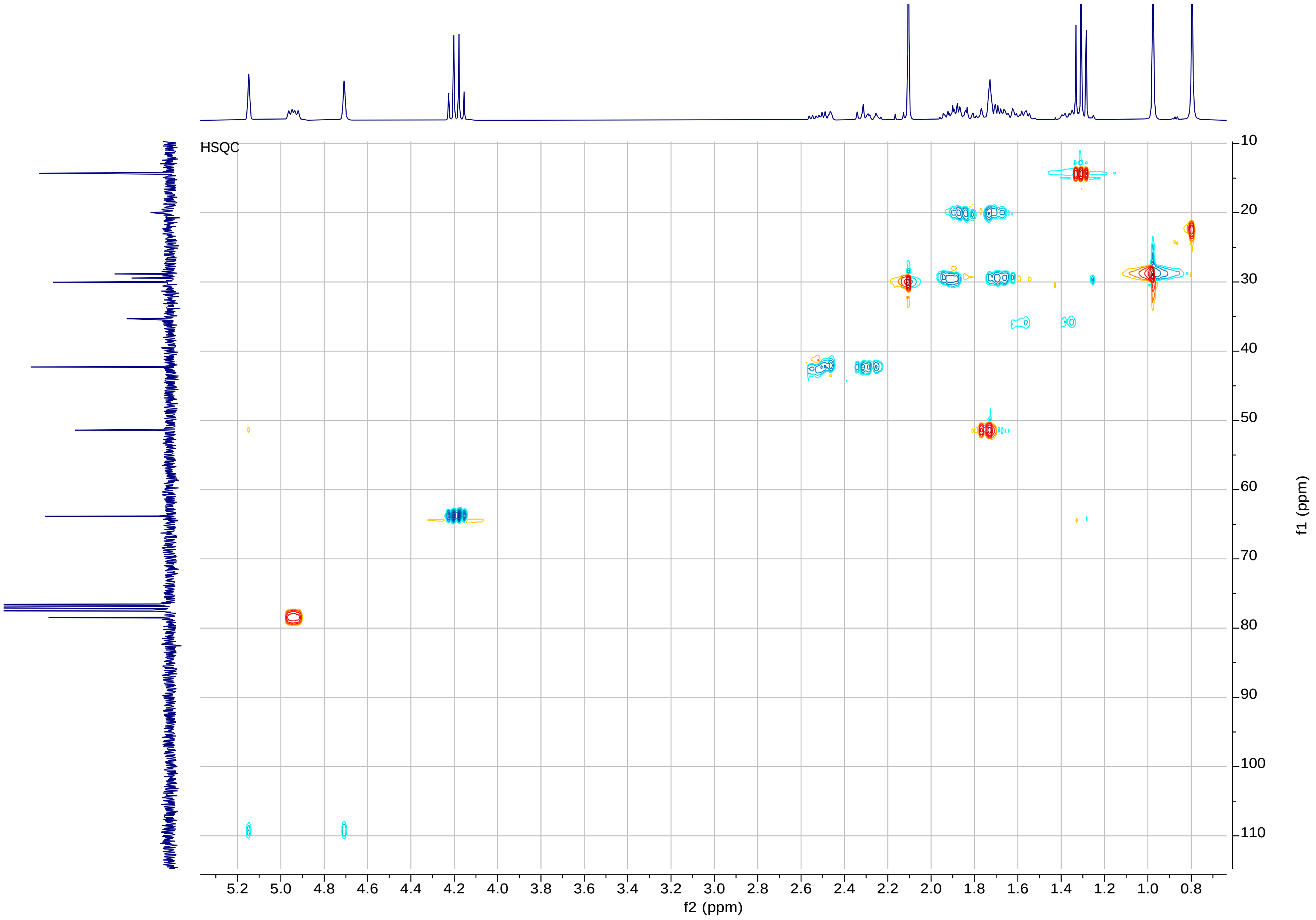
HSQC
5.2
5.0
4.8
4.6
4.4
4.2
4.0
3.8
3.6
3.4
3.2
3.0
2.8
2.6
2.4
2.2
2.0
1.8
1.6
1.4
1.2
1.0
0.8
f2 (ppm)
10
20
30
40
50
60
70
80
90
100
110
f1 (ppm)

Figure S20

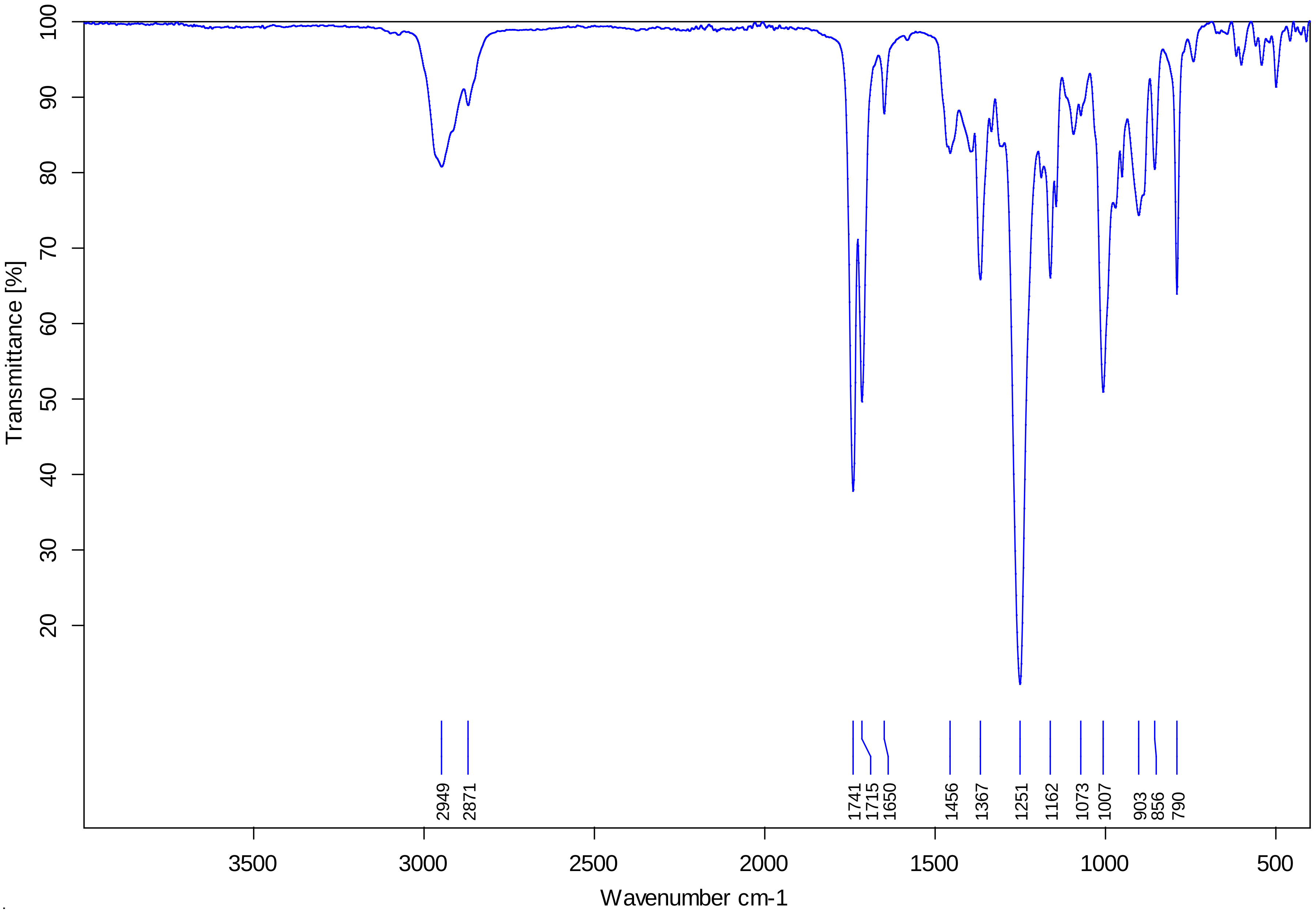
Transmittance [%]
100
90
80
70
60
50
40
30
20
Wavenumber cm-1
3500
3000
2500
2000
1500
1000
500
2949
2871
1741
1715
1650
1456
1367
1251
1162
1073
1007
903
856
790

Figure S21

^{1}H NMR, DEPT 135, ^{13}C NMR, HSQC, HMBC, COSY, NOESY 1D and IR of 6

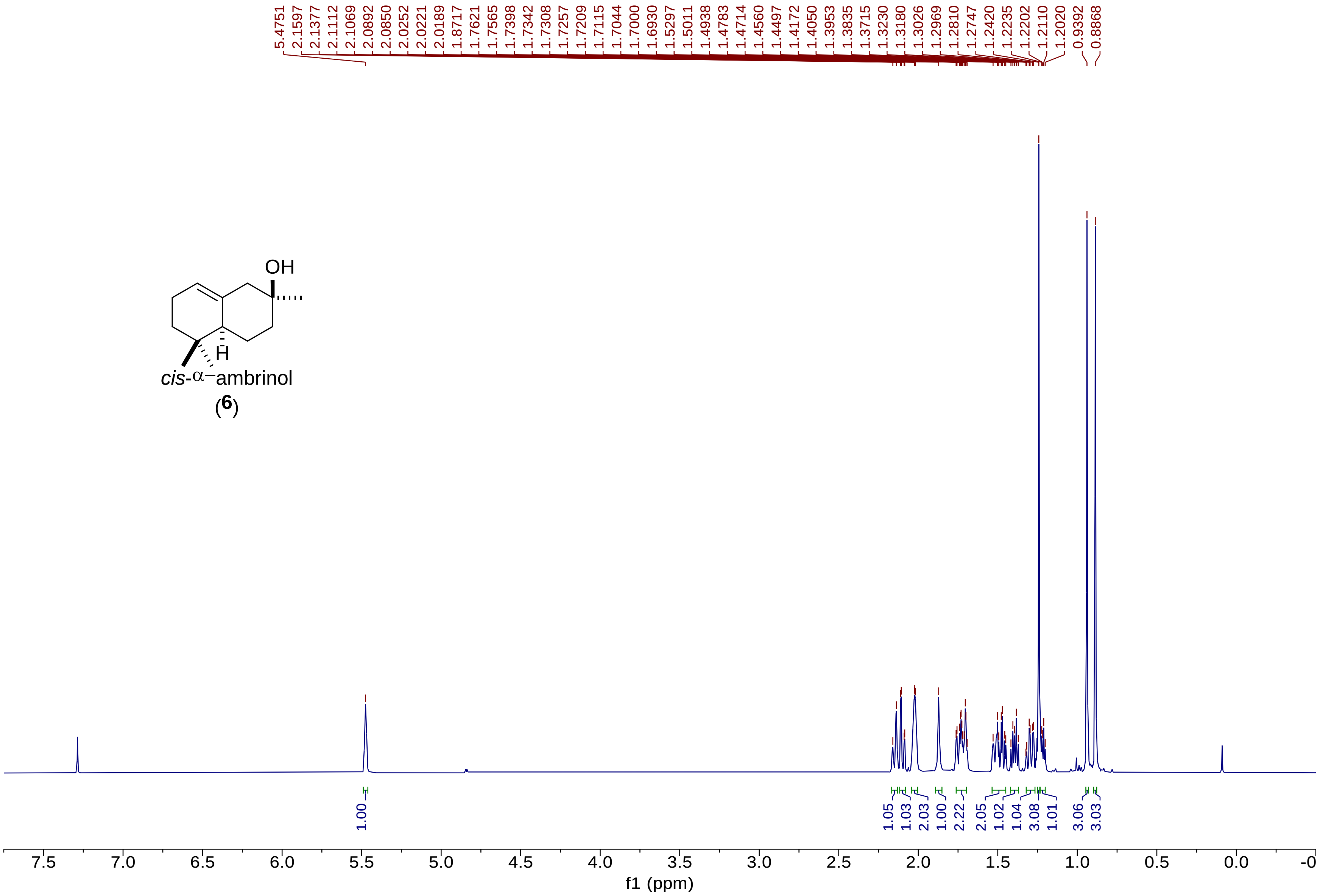

Figure S22

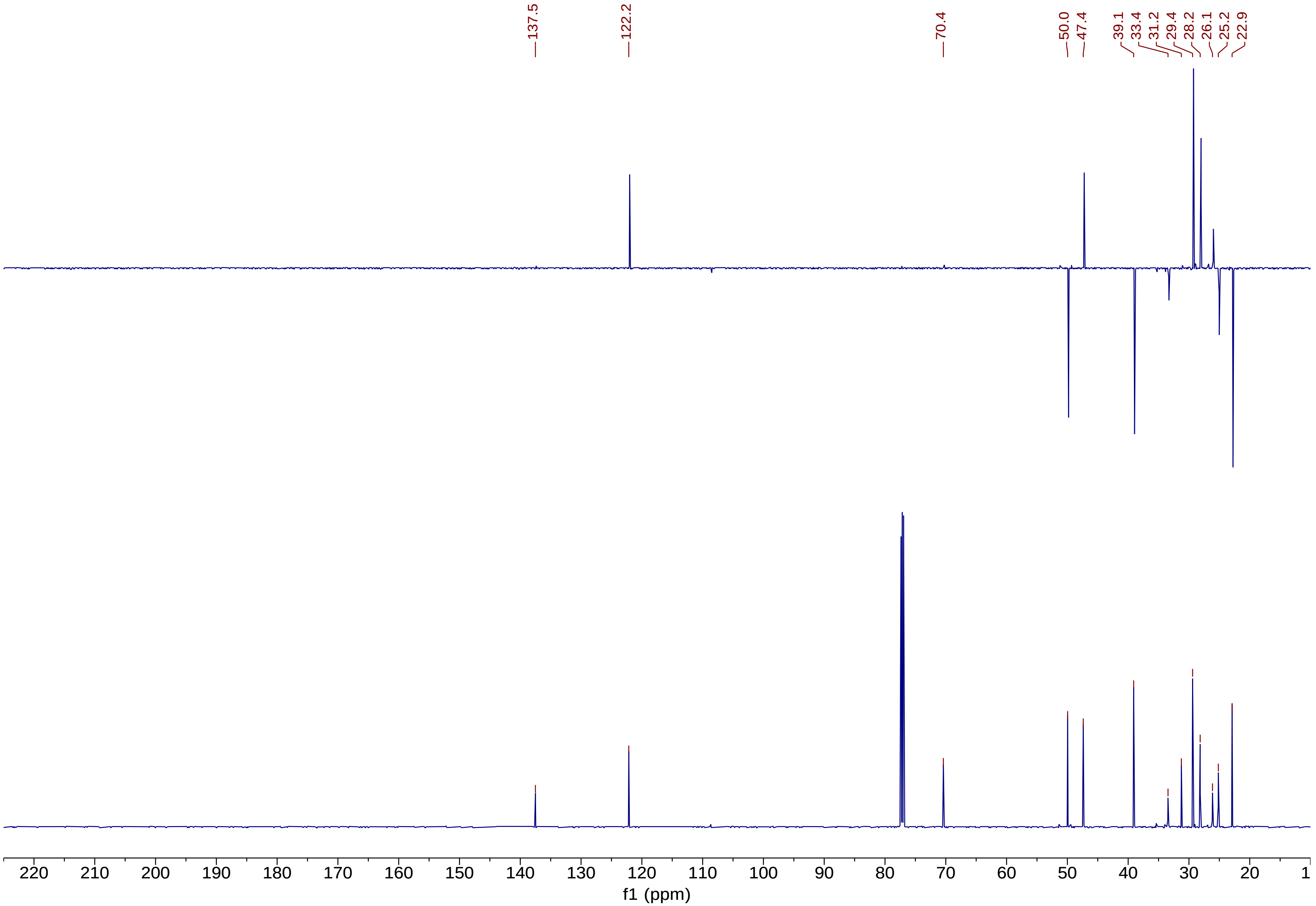

137.5
122.2
70.4
50.0
47.4
39.1
33.4
31.2
29.4
28.2
26.1
25.2
22.9
220
210
200
190
180
170
160
150
140
130
120
110
100
90
80
70
60
50
40
30
20
1
f1 (ppm)

Figure S23

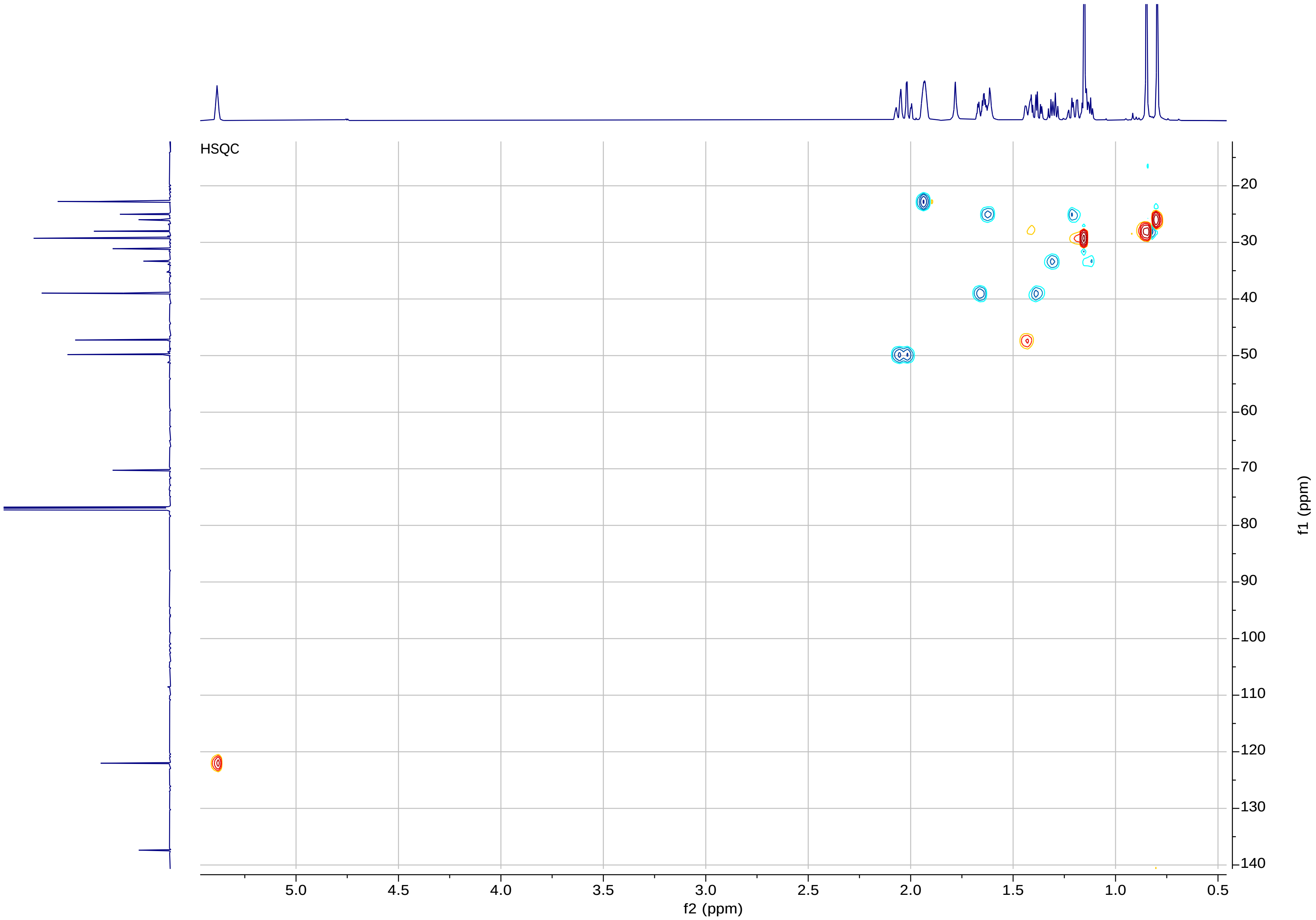
HSQC
f2 (ppm)
f1 (ppm)
5.0
4.5
4.0
3.5
3.0
2.5
2.0
1.5
1.0
0.5
20
30
40
50
60
70
80
90
100
110
120
130
140

Figure S24

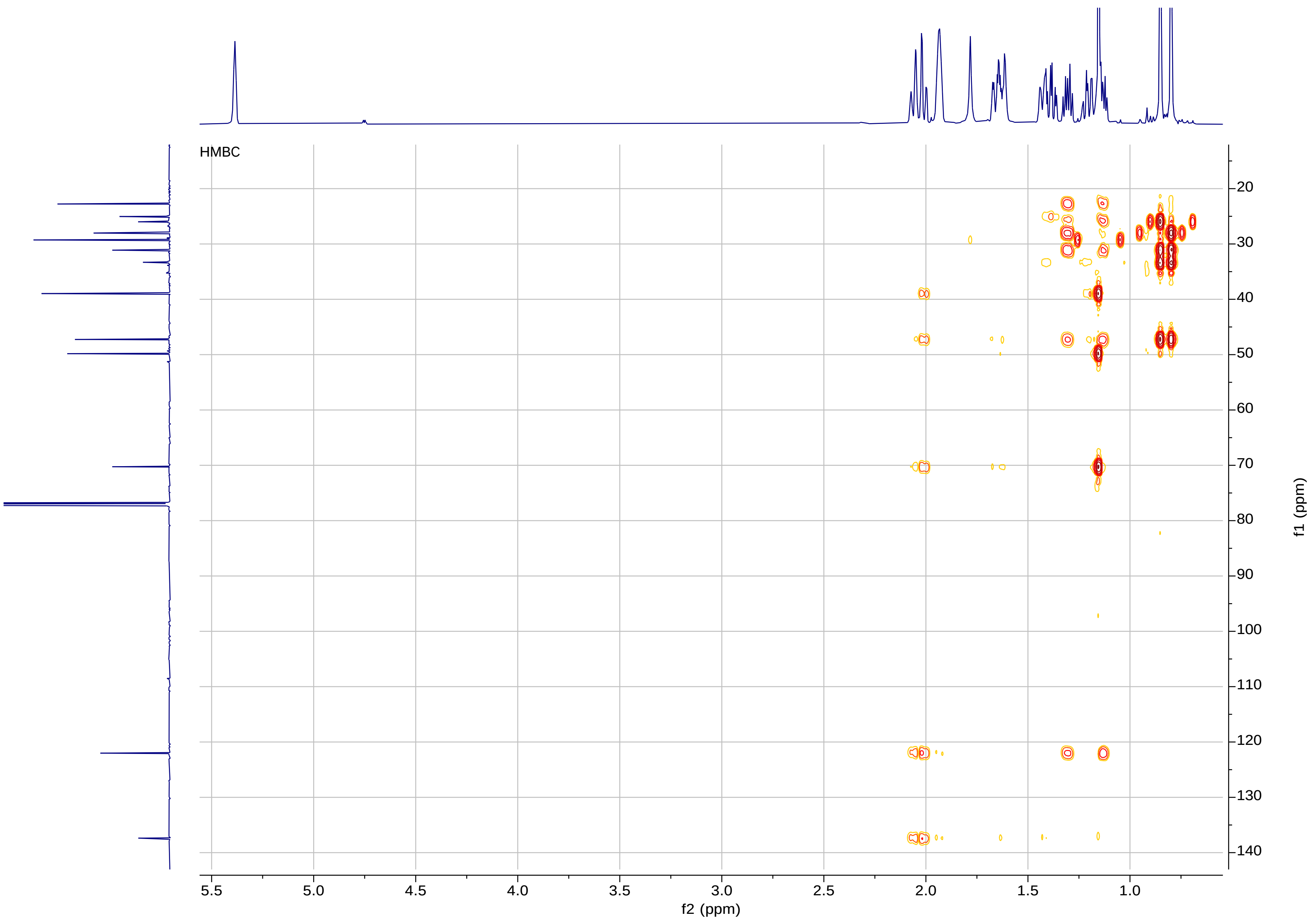
HMBC
f2 (ppm)
f1 (ppm)
5.5
5.0
4.5
4.0
3.5
3.0
2.5
2.0
1.5
1.0
20
30
40
50
60
70
80
90
100
110
120
130
140

Figure S25

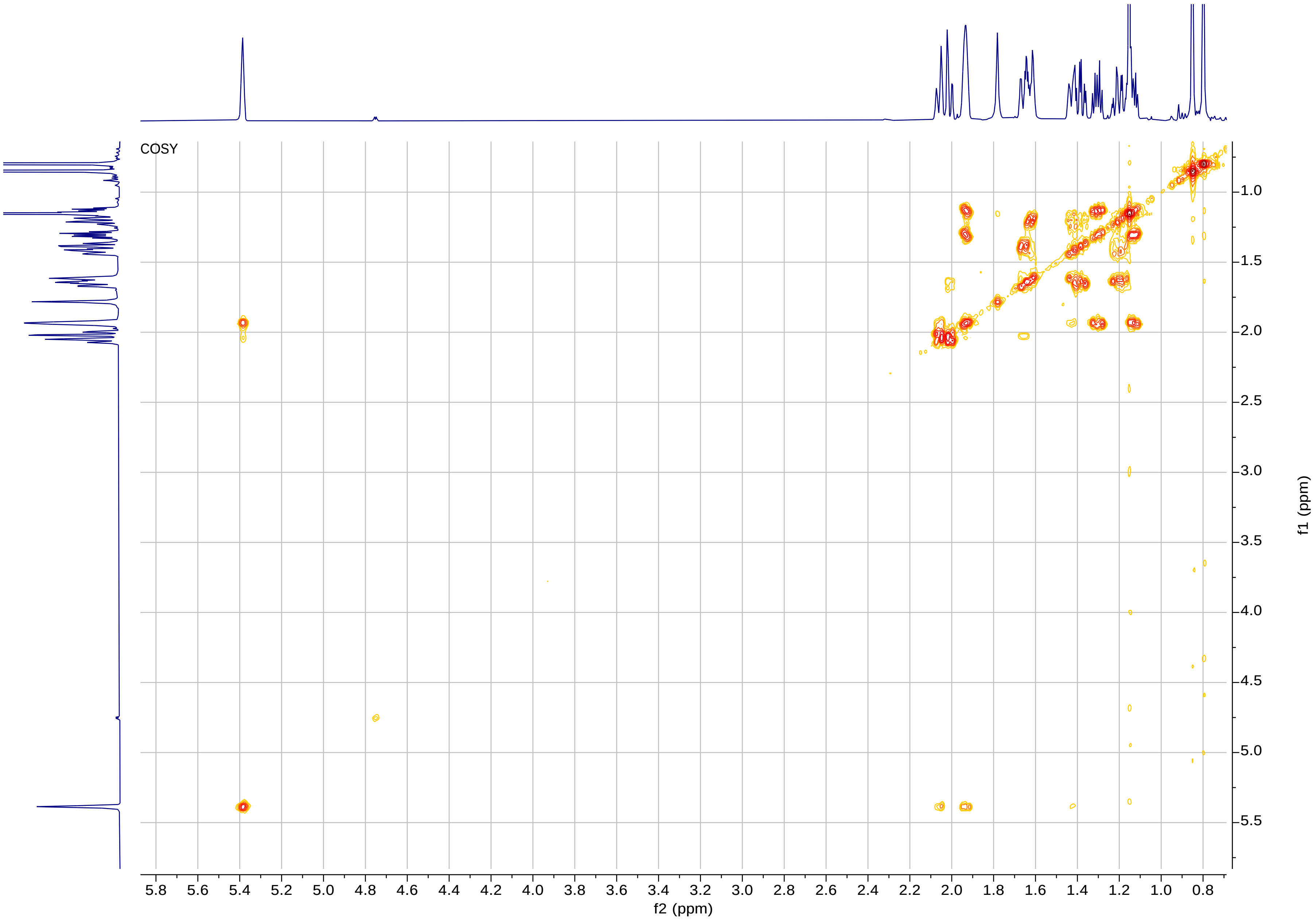
COSY
f2 (ppm)
f1 (ppm)
5.8
5.6
5.4
5.2
5.0
4.8
4.6
4.4
4.2
4.0
3.8
3.6
3.4
3.2
3.0
2.8
2.6
2.4
2.2
2.0
1.8
1.6
1.4
1.2
1.0
0.8
1.0
1.5
2.0
2.5
3.0
3.5
4.0
4.5
5.0
5.5

Figure S26

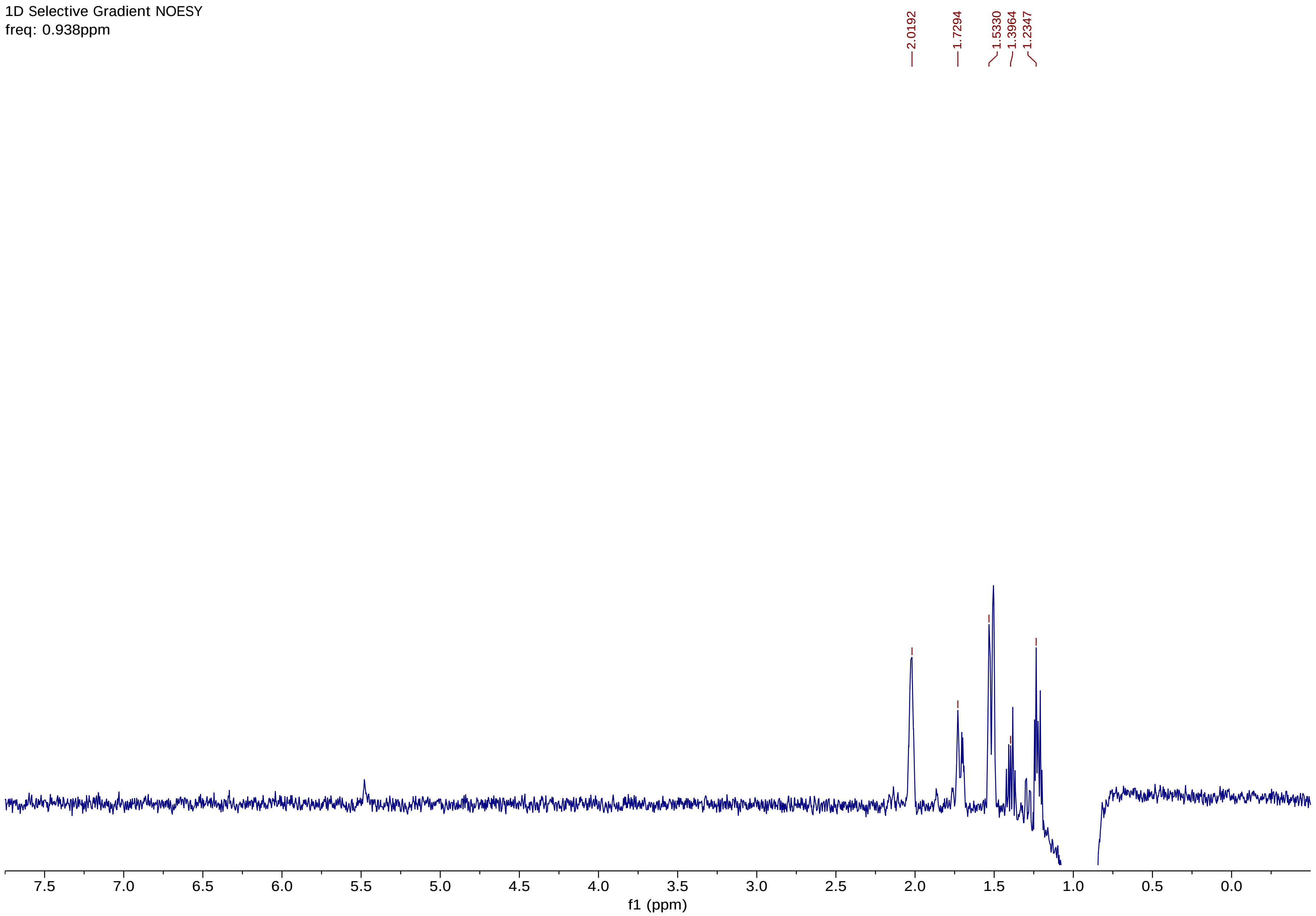
1D Selective Gradient NOESY
freq: 0.938ppm
2.0192
1.7294
1.5330
1.3964
1.2347
7.5
7.0
6.5
6.0
5.5
5.0
4.5
4.0
3.5
3.0
2.5
2.0
1.5
1.0
0.5
0.0
f1 (ppm)

Figure S27

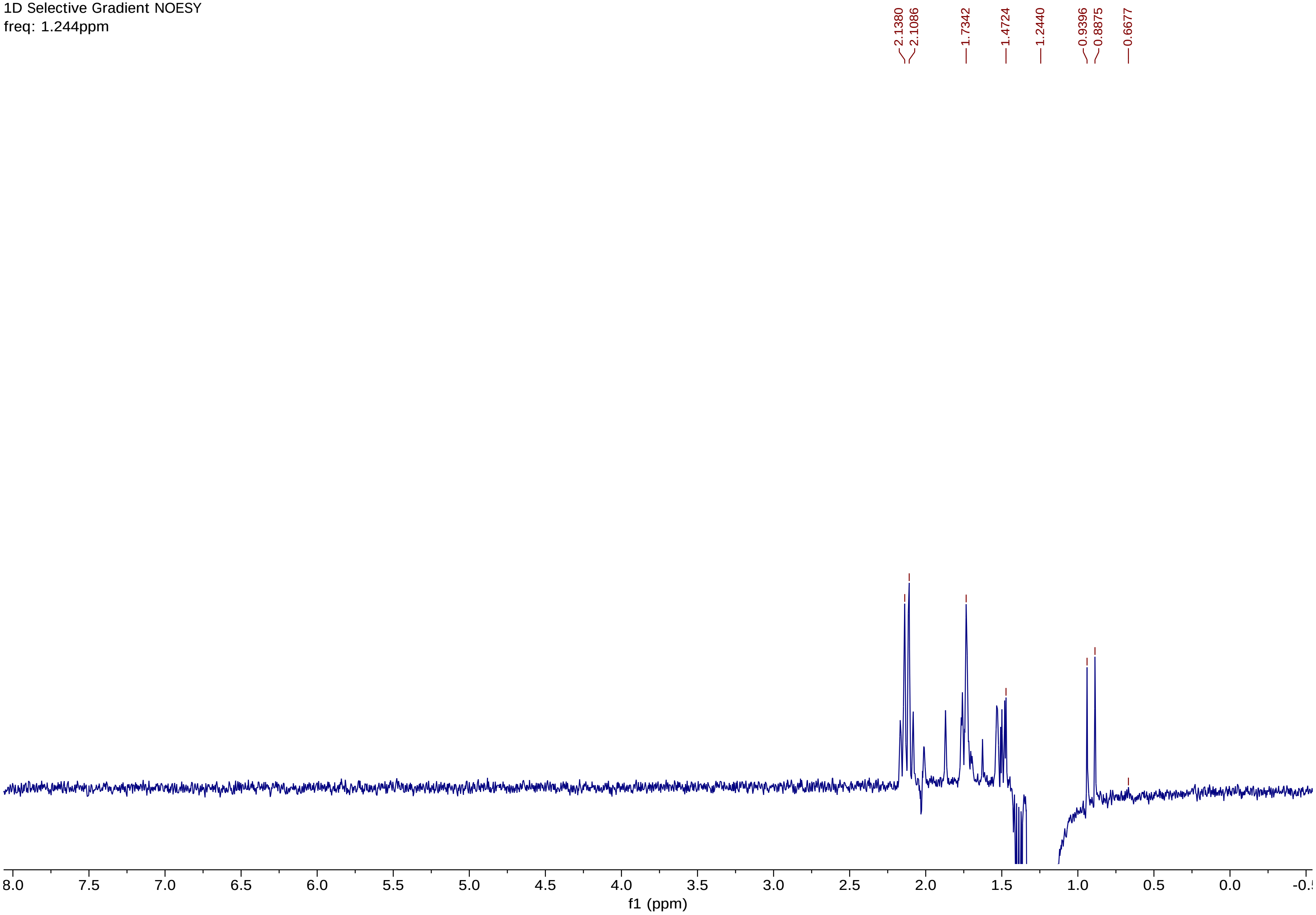
1D Selective Gradient NOESY
freq: 1.244ppm
2.1380
2.1086
1.7342
1.4724
1.2440
0.9396
0.8875
0.6677
8.0
7.5
7.0
6.5
6.0
5.5
5.0
4.5
4.0
3.5
3.0
2.5
2.0
1.5
1.0
0.5
0.0
f1 (ppm)

Figure S28

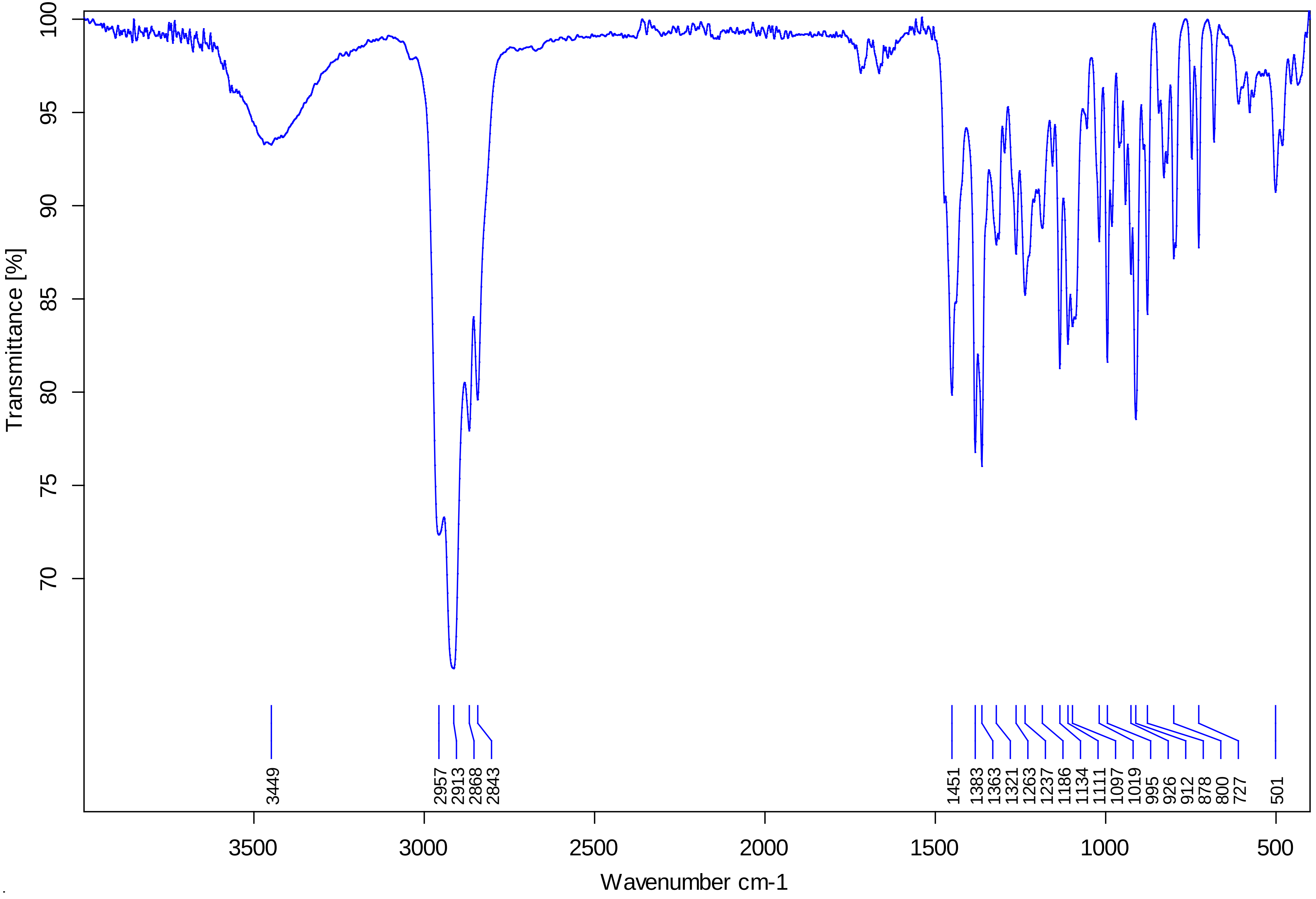

Transmittance [%]
100
95
90
85
80
75
70
3449
2957
2913
2868
2843
1451
1383
1363
1321
1263
1237
1186
1134
1111
1097
1019
995
926
912
878
800
727
501
3500
3000
2500
2000
1500
1000
500
Wavenumber cm-1

Figure S29

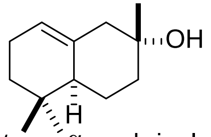

trans-α-ambrinol
(**24**)

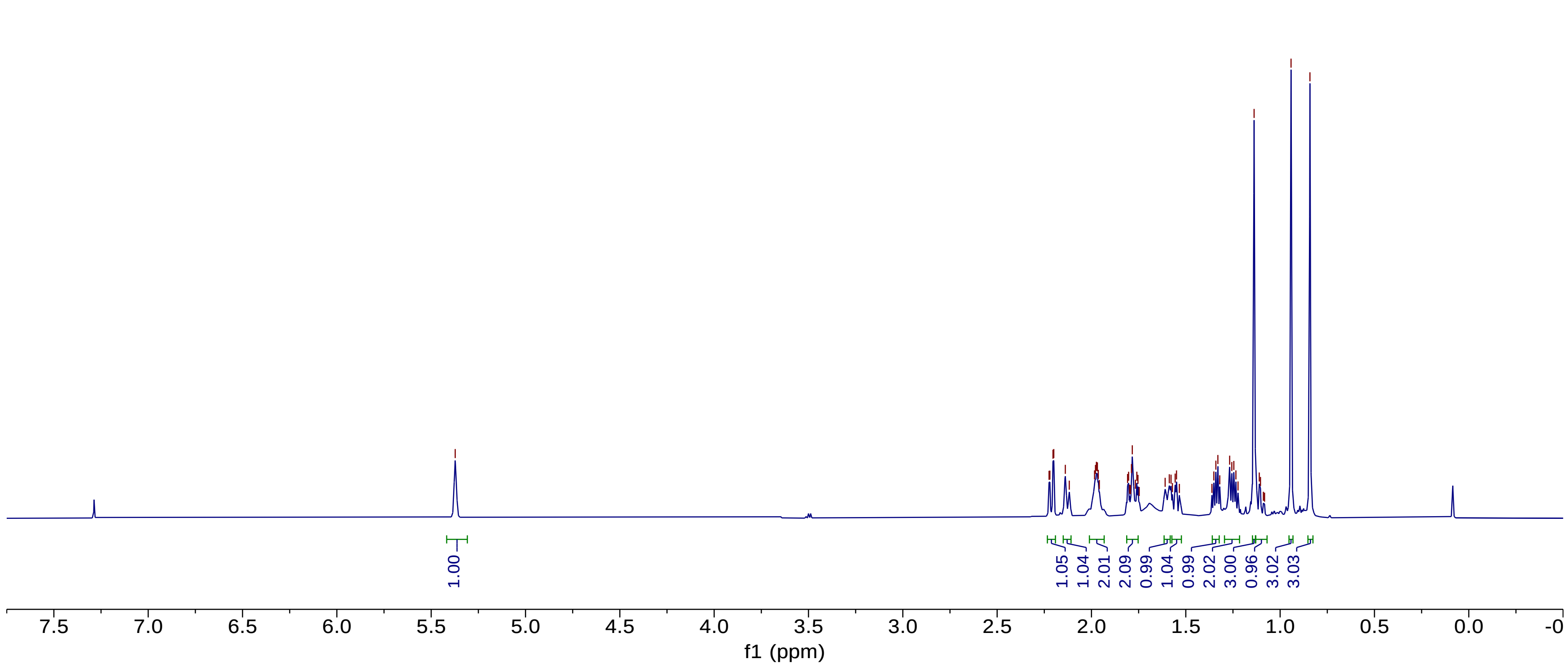

Figure S30

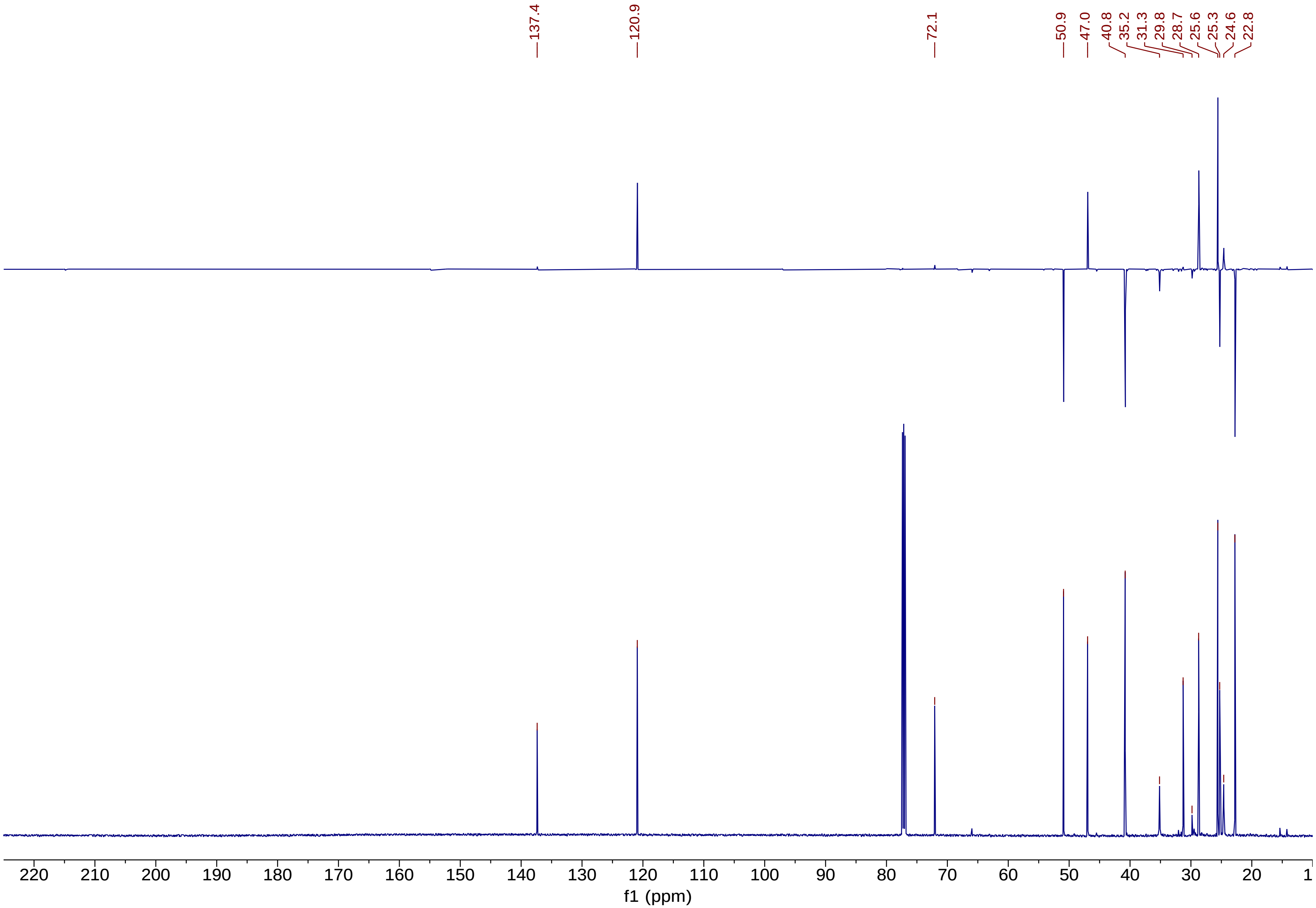
137.4
120.9
72.1
50.9
47.0
40.8
35.2
31.3
29.8
28.7
25.6
25.3
24.6
22.8
220
210
200
190
180
170
160
150
140
130
120
110
100
90
80
70
60
50
40
30
20
1
f1 (ppm)

Figure S31

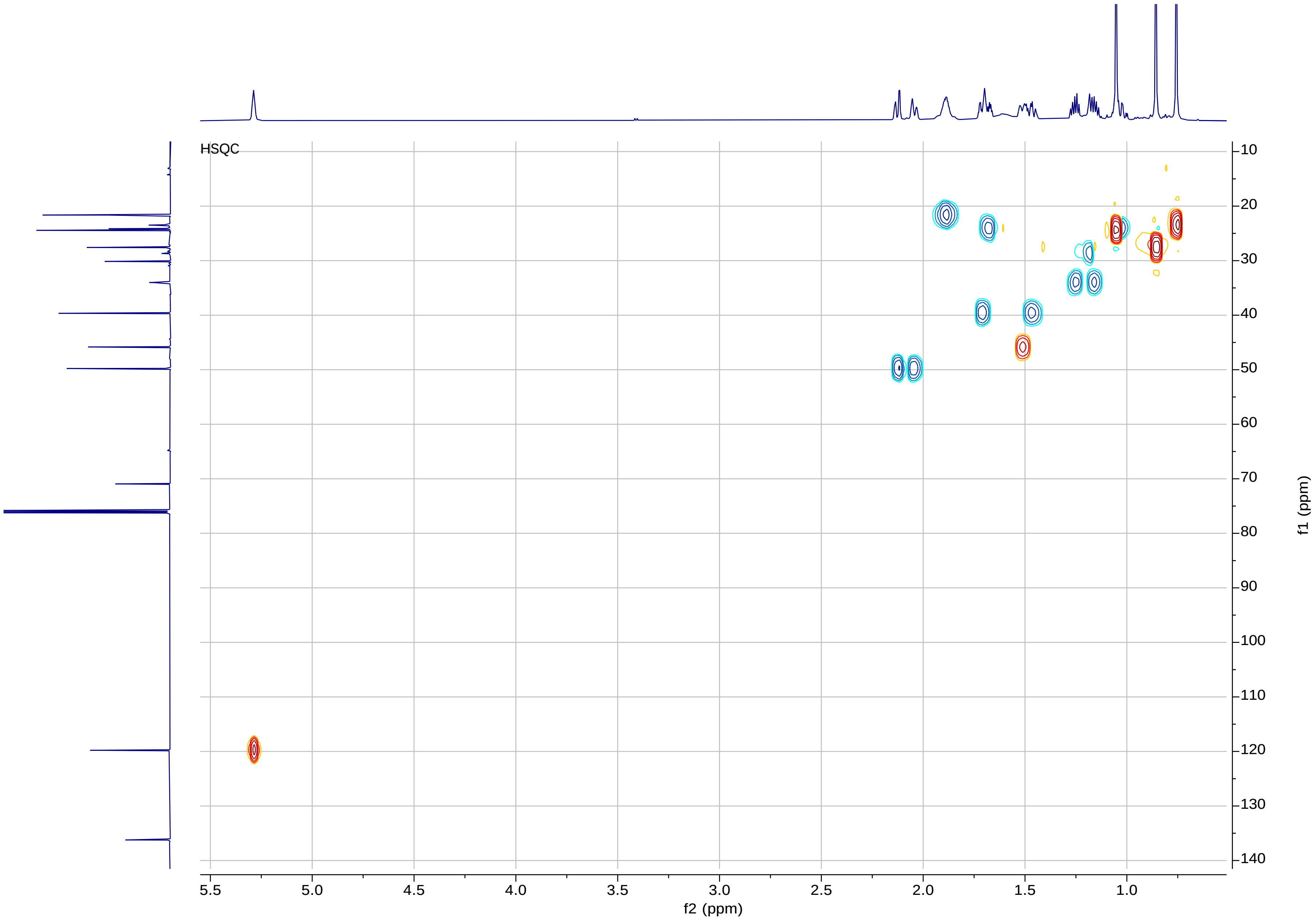

Figure S32

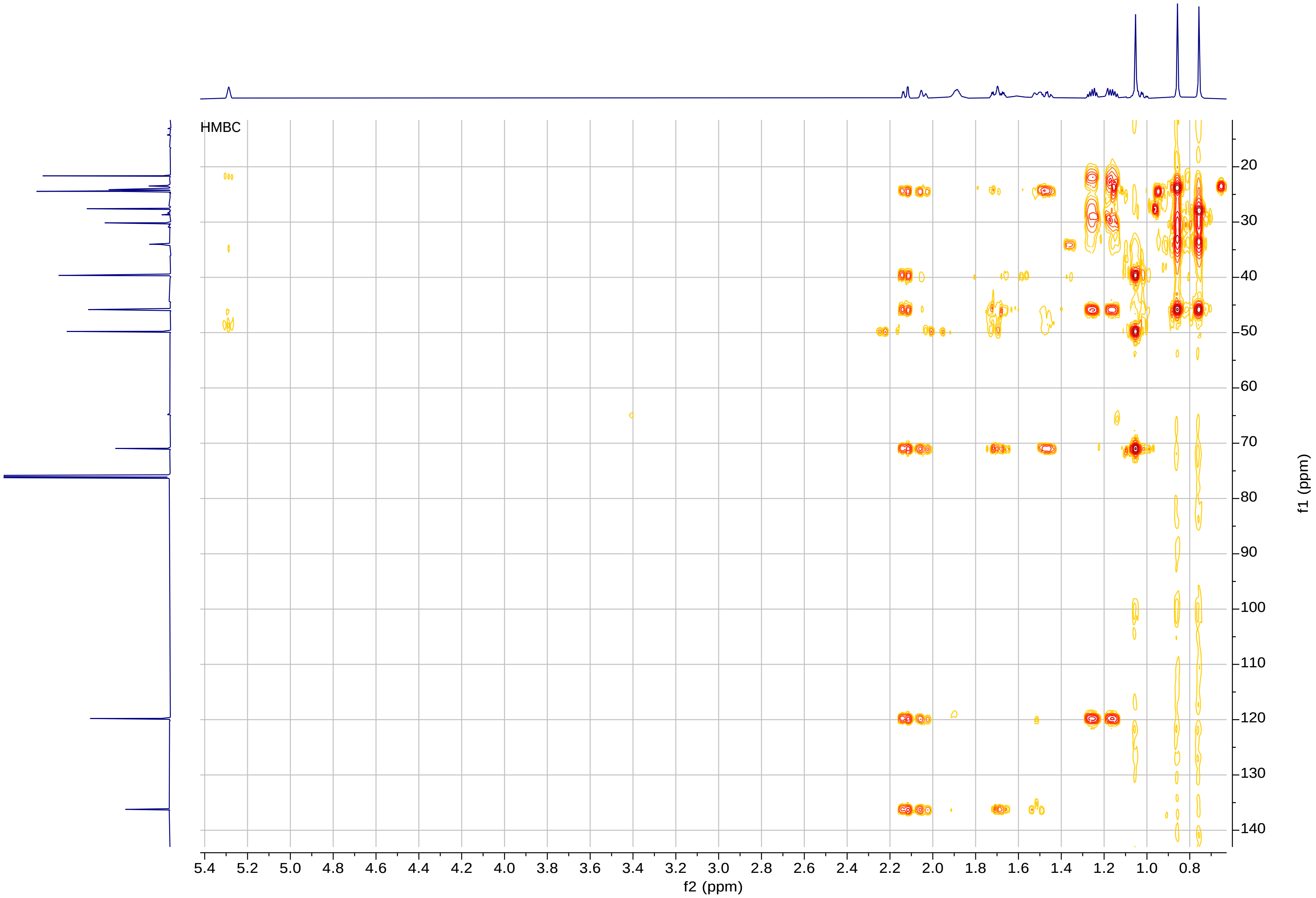

Figure S33

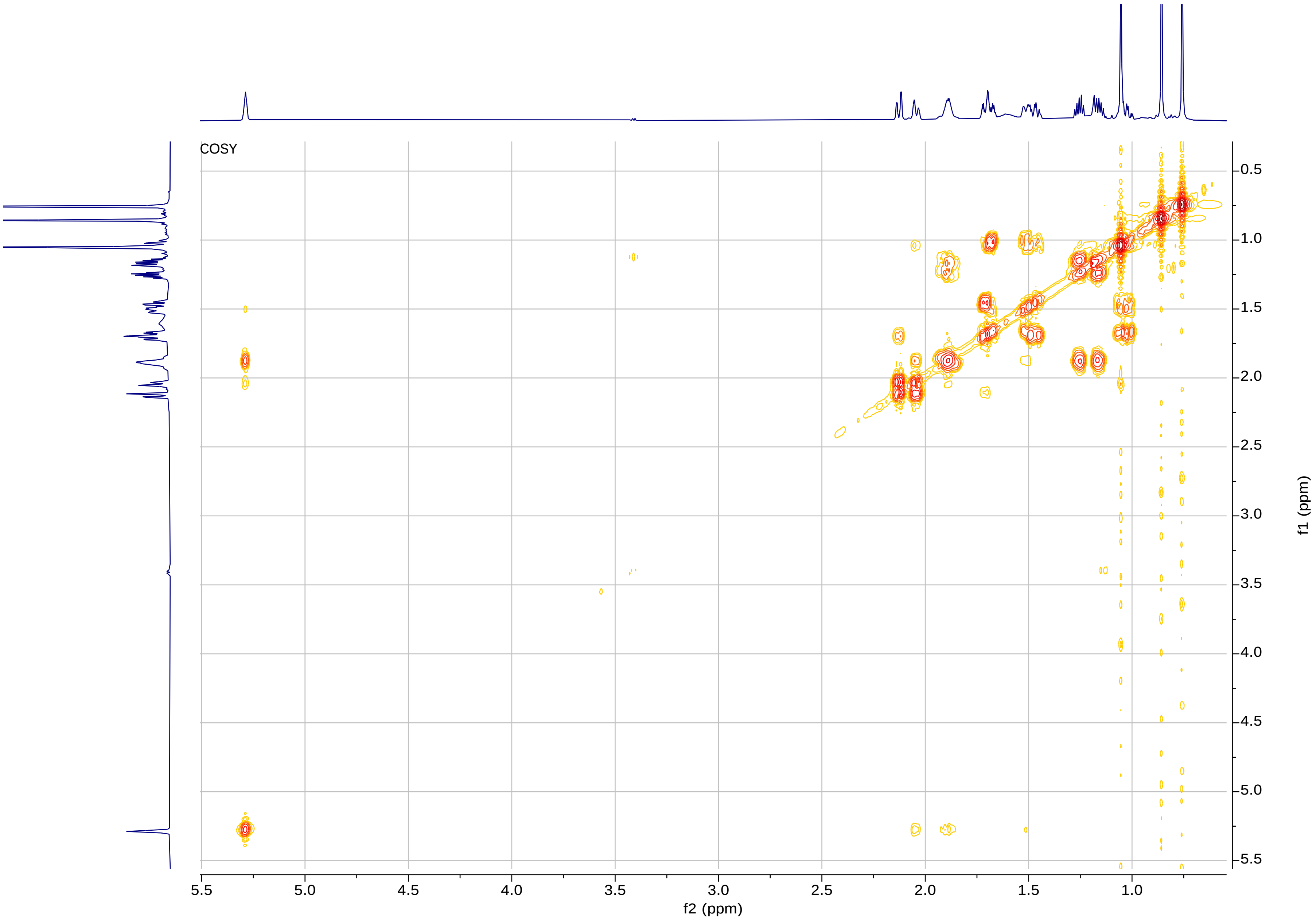

Figure S34

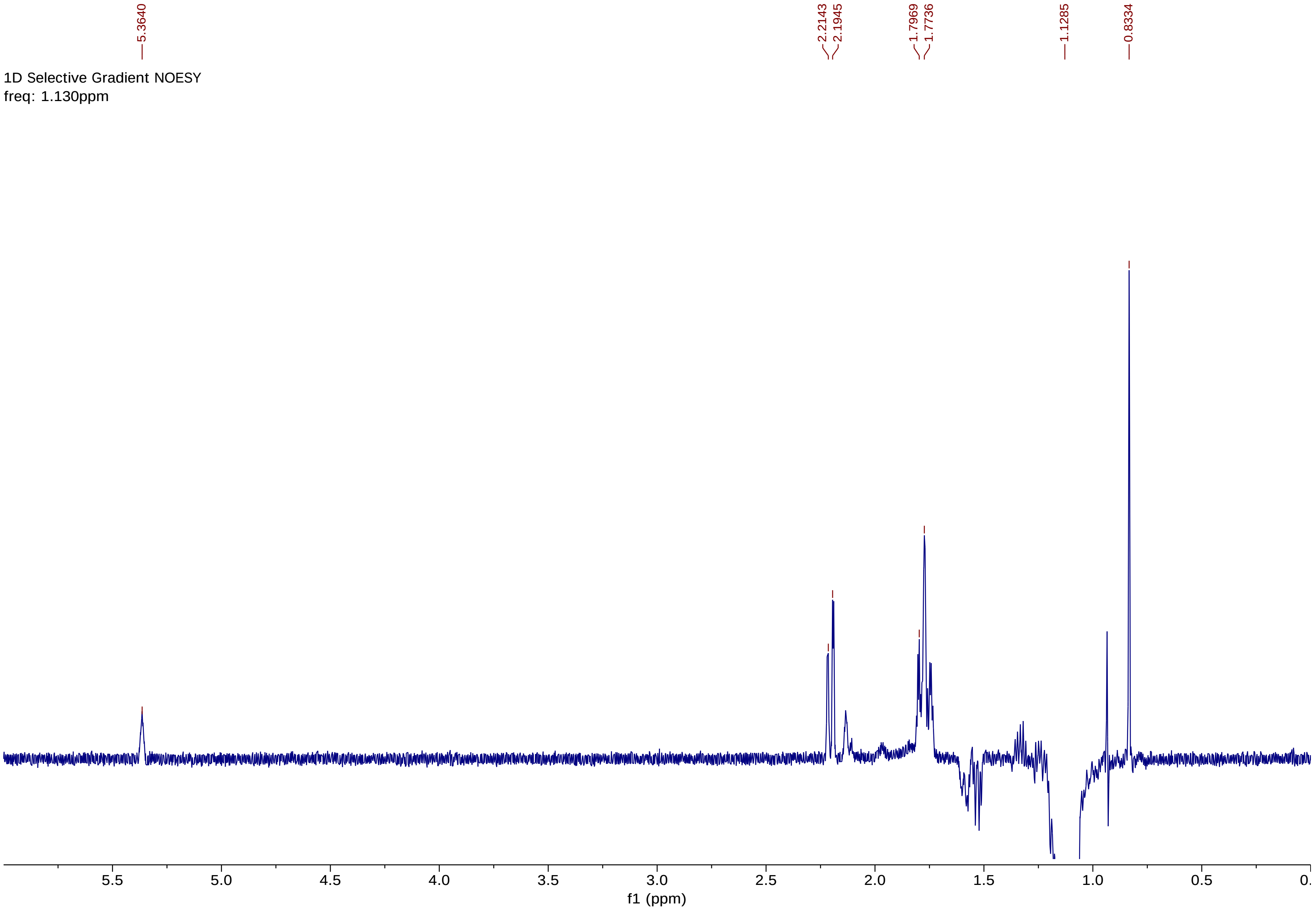

5.3640
2.2143
2.1945
1.7969
1.7736
1.1285
0.8334
1D Selective Gradient NOESY
freq: 1.130ppm
5.5
5.0
4.5
4.0
3.5
3.0
2.5
2.0
1.5
1.0
0.5
f1 (ppm)

Figure S35

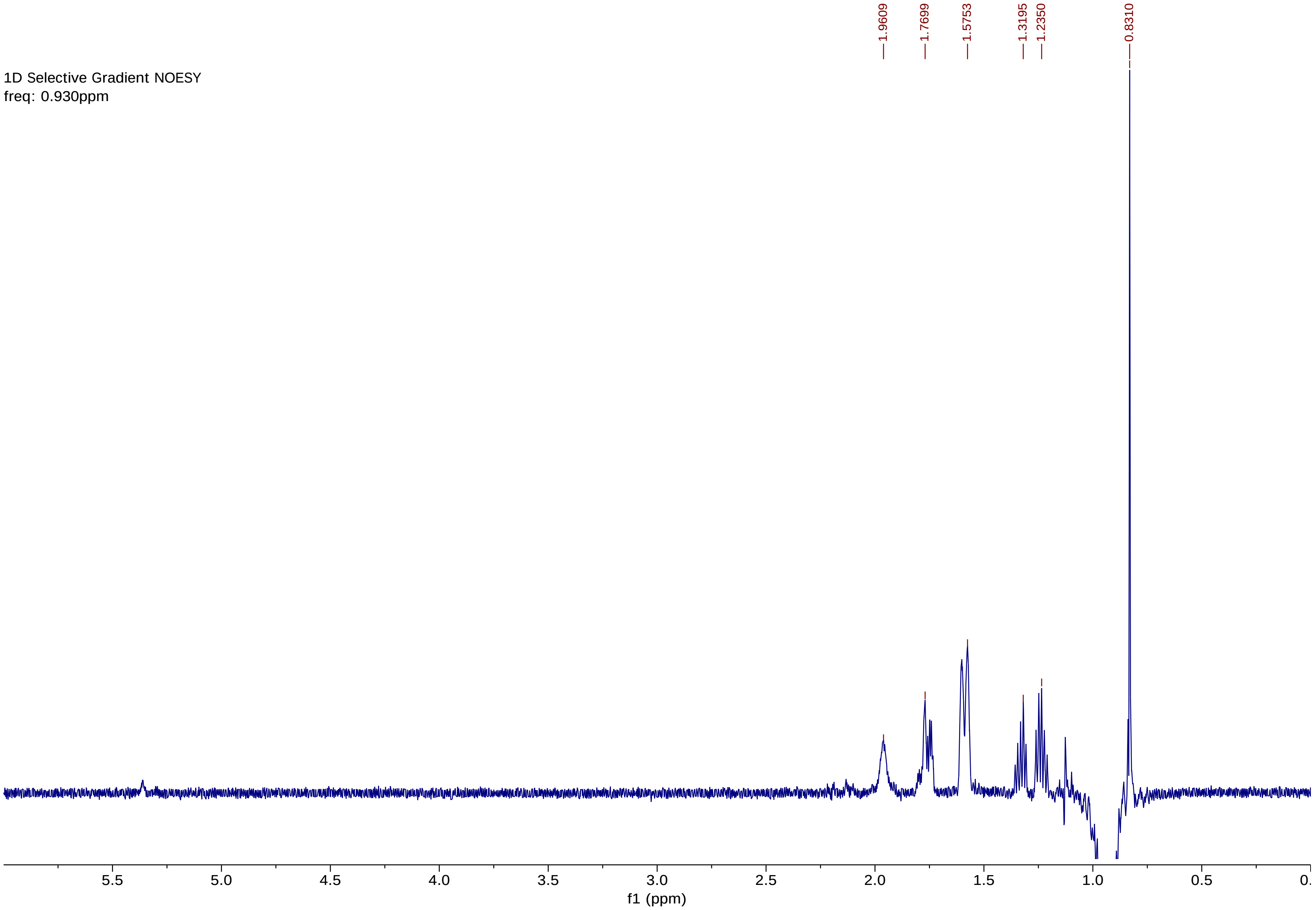
1.9609
1.7699
1.5753
1.3195
1.2350
0.8310
1D Selective Gradient NOESY
freq: 0.930ppm
5.5
5.0
4.5
4.0
3.5
3.0
2.5
2.0
1.5
1.0
0.5
f1 (ppm)

Figure S36

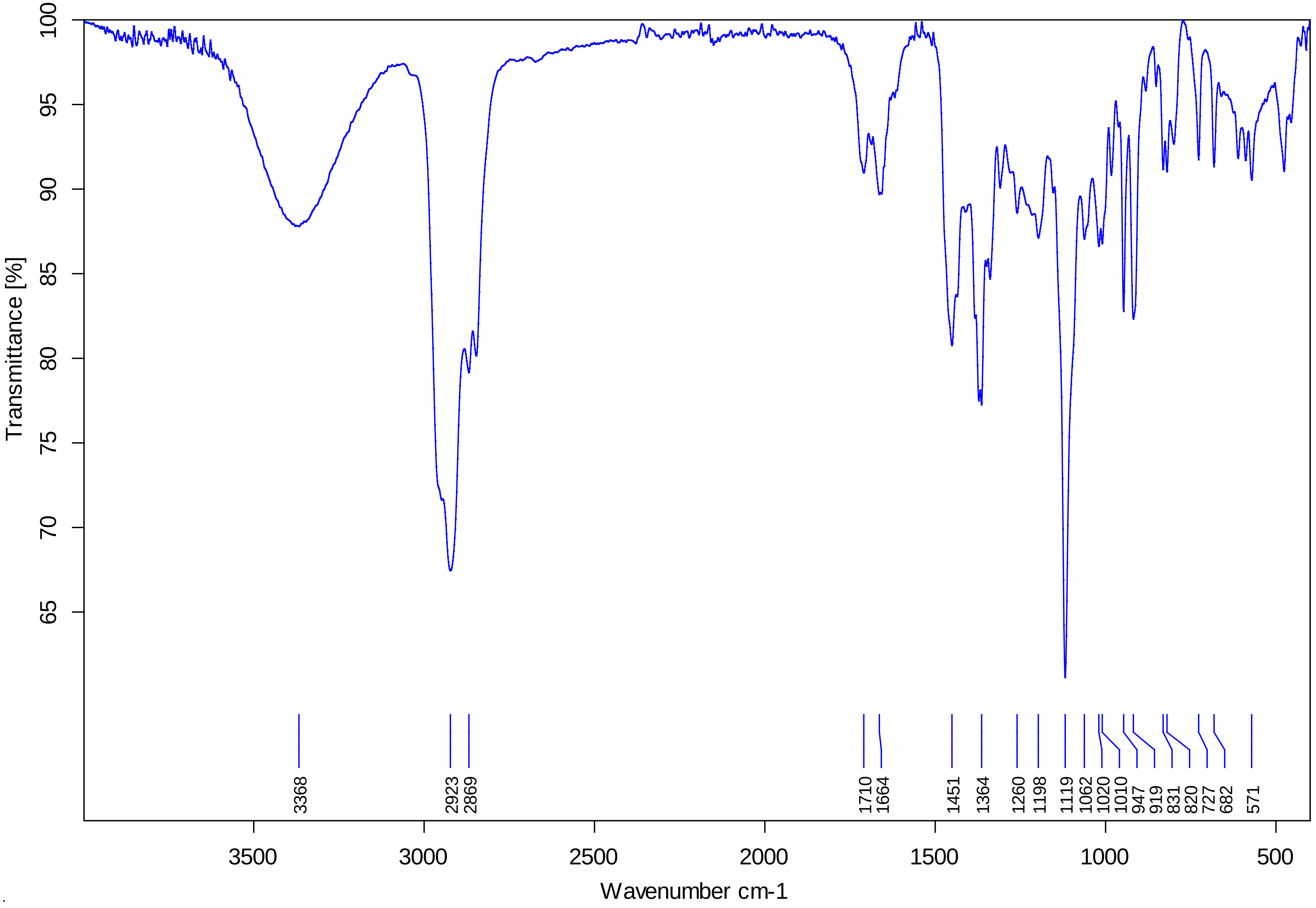

Transmittance [%]
100
95
90
85
80
75
70
65
3368
2923
2869
1710
1664
1451
1364
1260
1198
1119
1062
1020
1010
947
919
831
820
727
682
571
3500
3000
2500
2000
1500
1000
500
Wavenumber cm-1

Figure S37

^{1}H NMR of 25

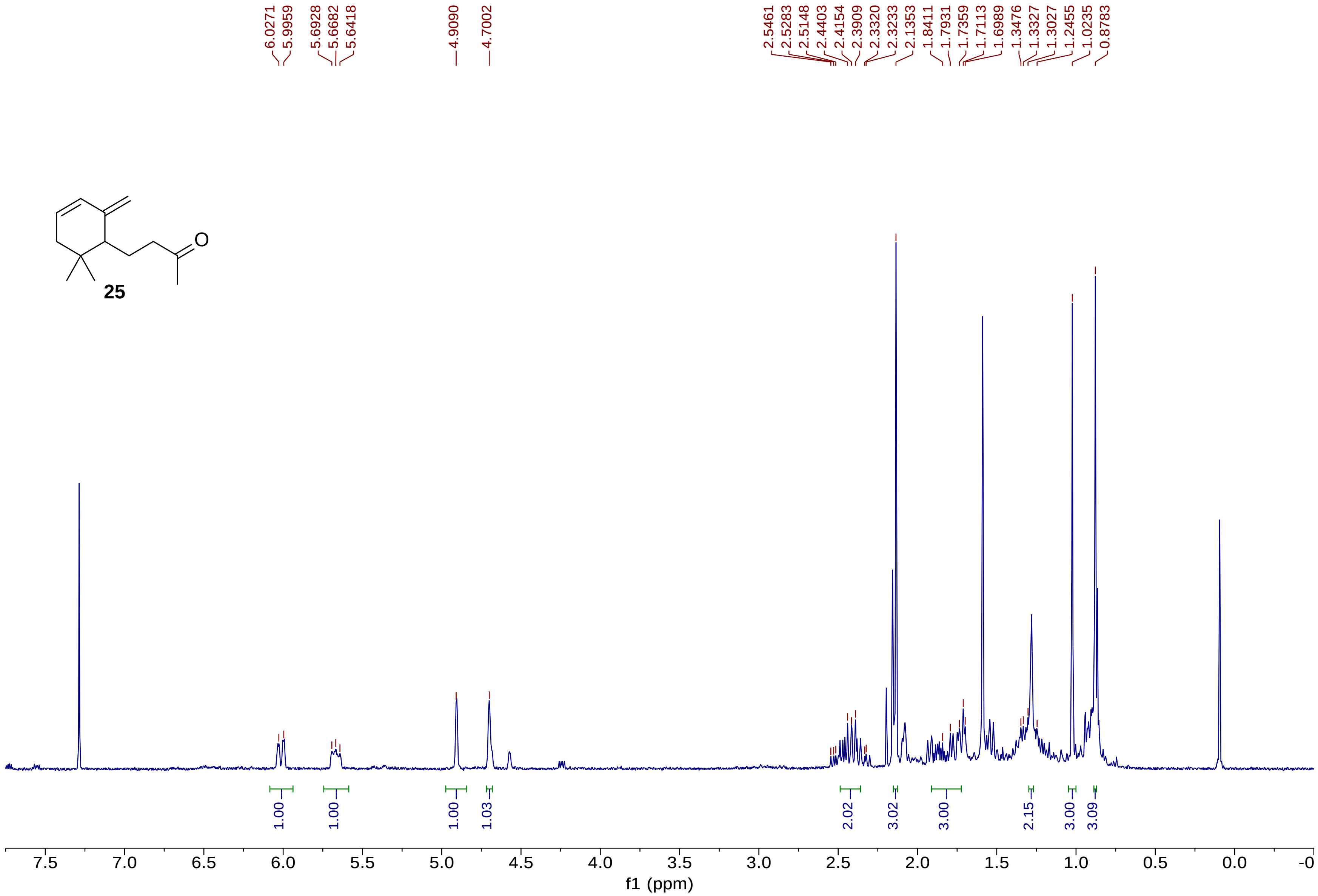

Figure S38

^{1}H NMR, DEPT 135, ^{13}C NMR and IR of 22

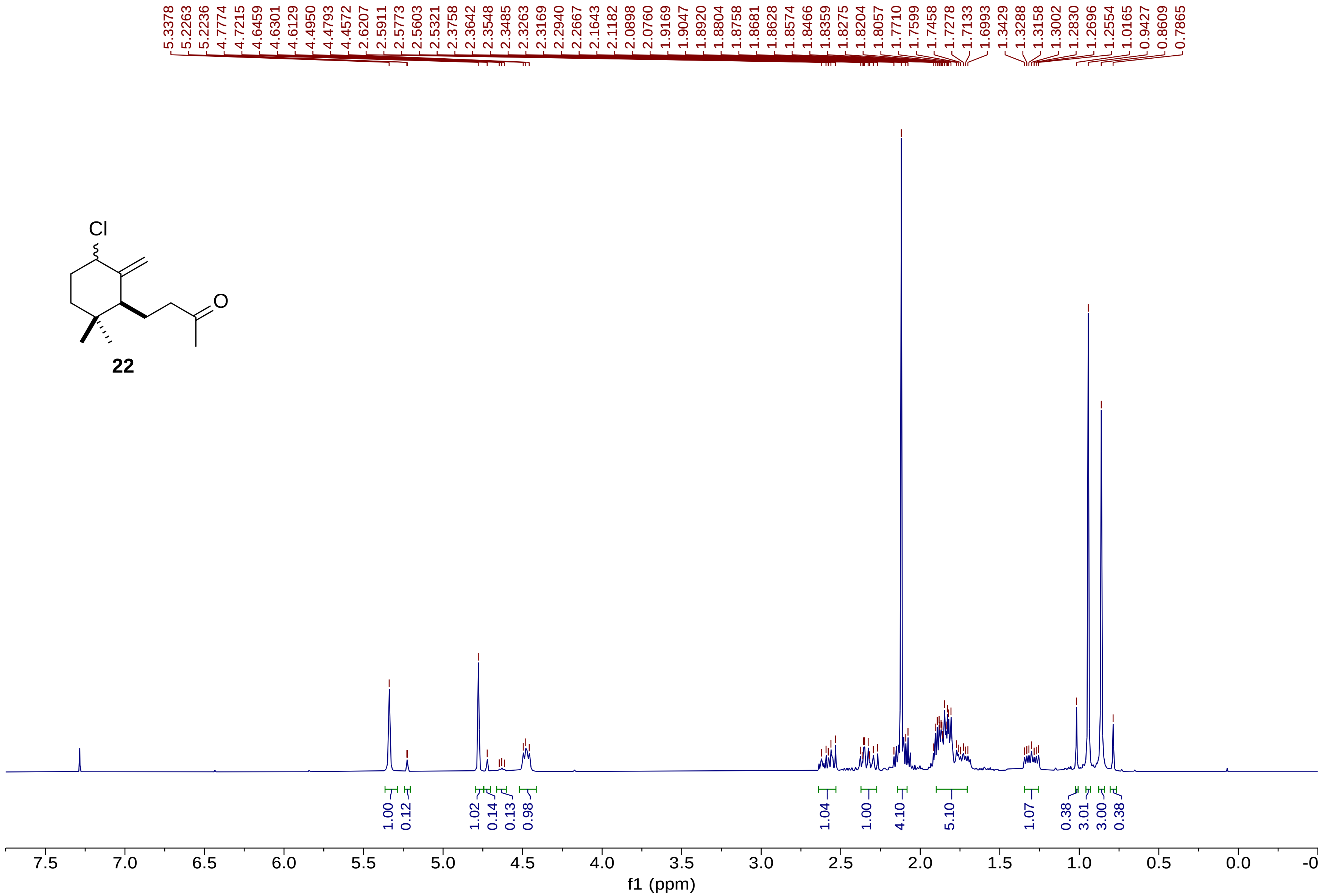

Figure S39

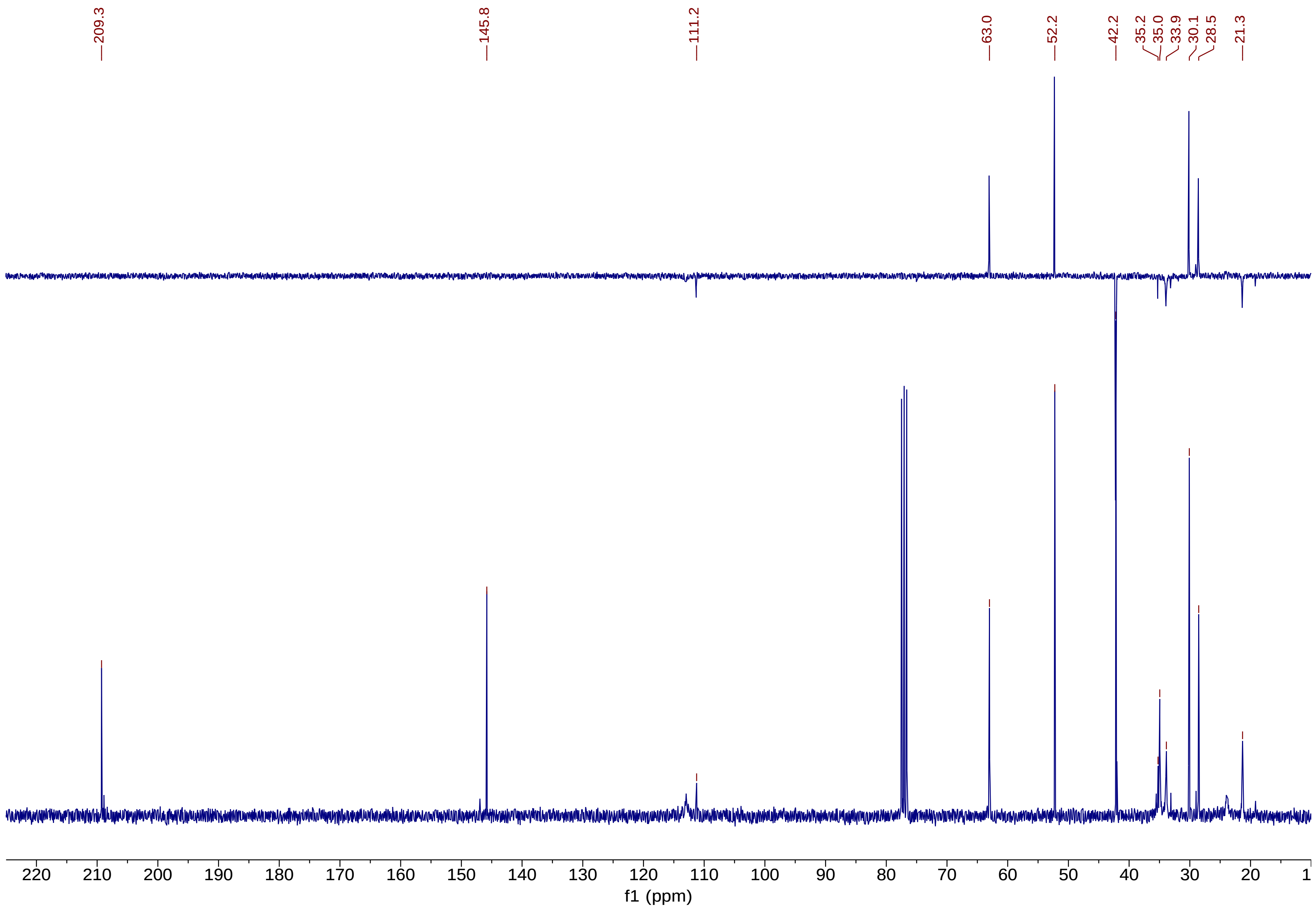
209.3
145.8
111.2
63.0
52.2
42.2
35.2
35.0
33.9
30.1
28.5
21.3
220
210
200
190
180
170
160
150
140
130
120
110
100
90
80
70
60
50
40
30
20
f1 (ppm)

Figure S40

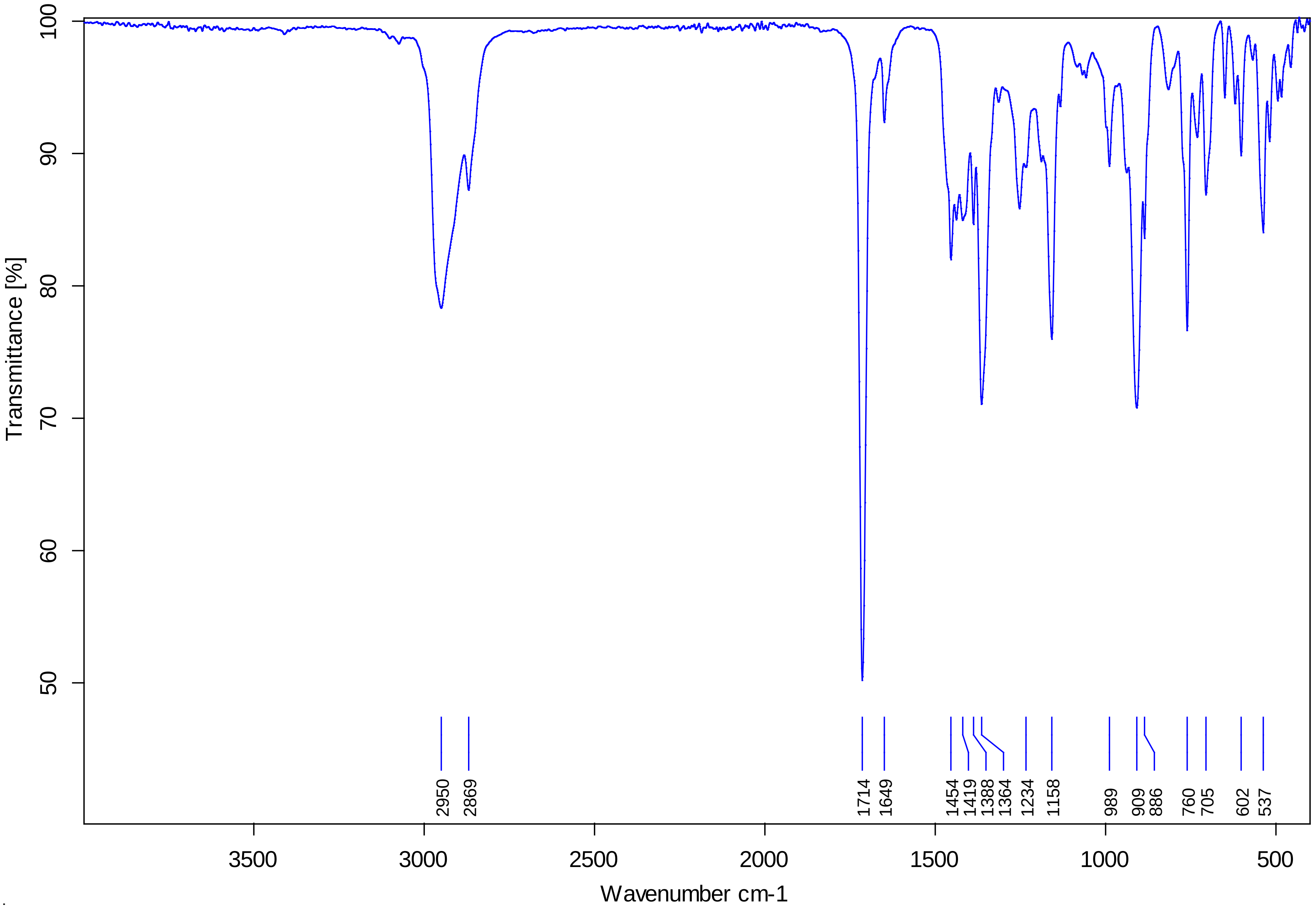
Transmittance [%]
100
90
80
70
60
50
2950
2869
1714
1649
1454
1419
1388
1364
1234
1158
989
909
886
760
705
602
537
3500
3000
2500
2000
1500
1000
500
Wavenumber cm-1

Figure S41

^{1}H NMR, DEPT 135, ^{13}C NMR, HSQC, HMBC, COSY and IR of 26

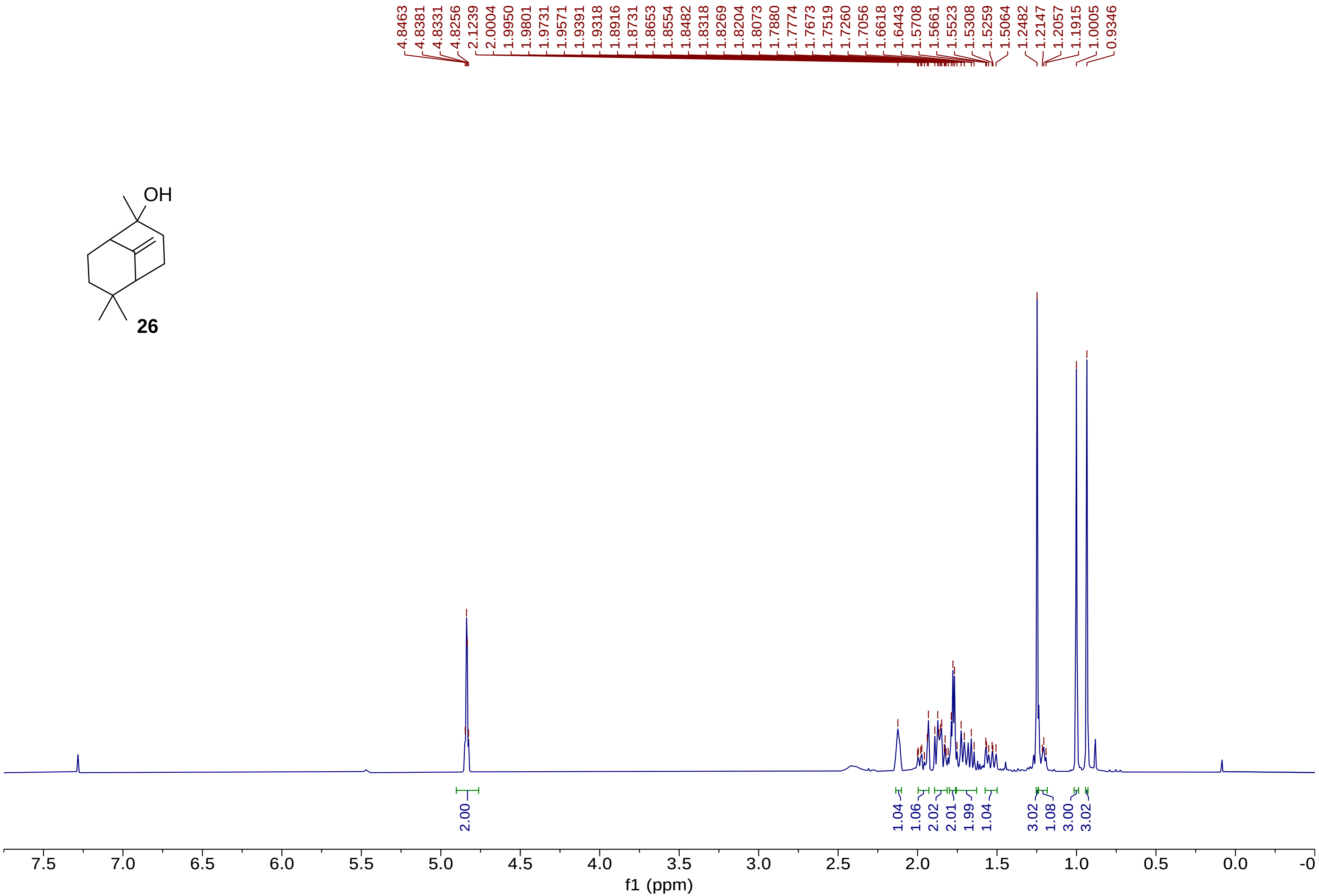

Figure S42

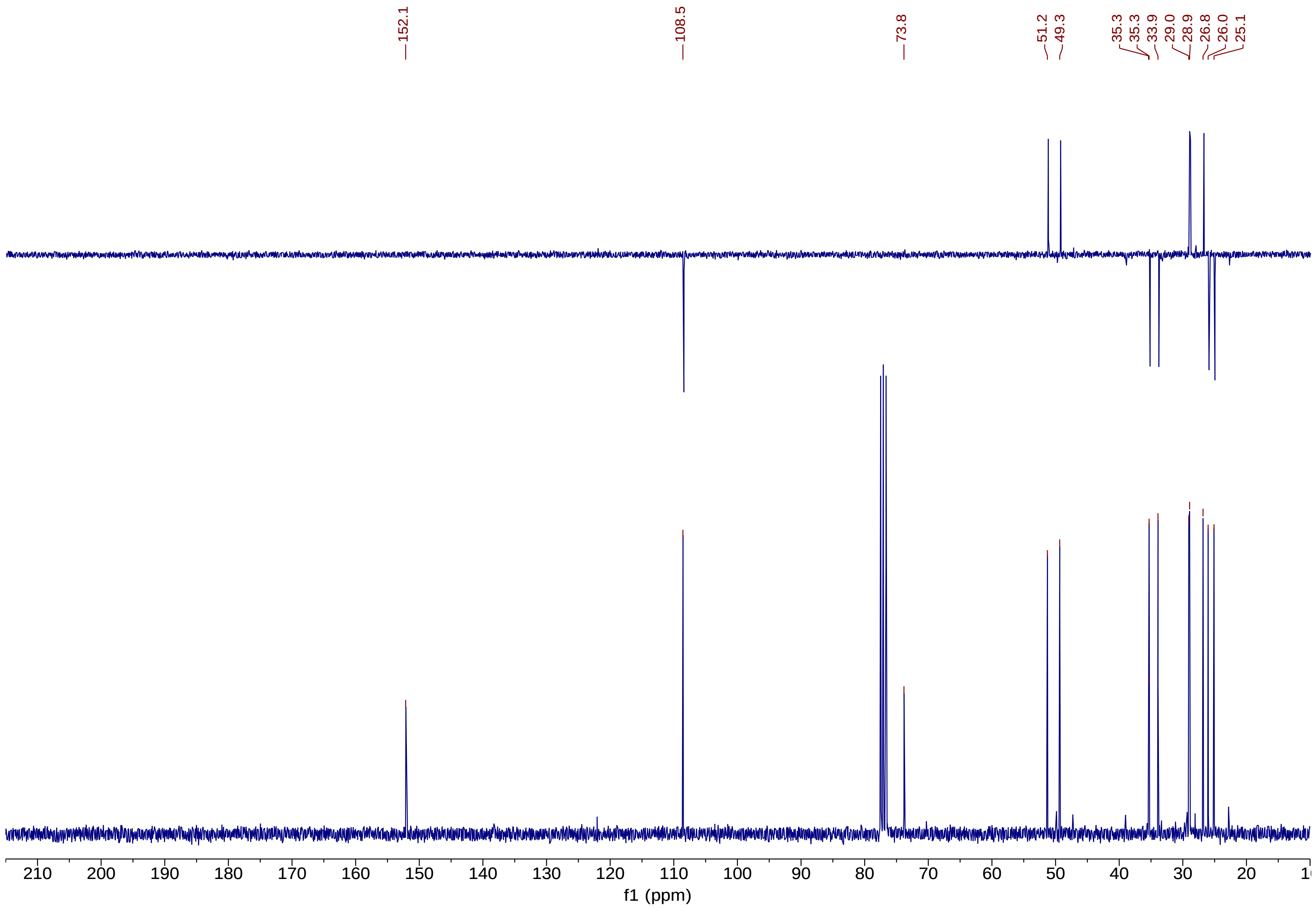

152.1
108.5
73.8
51.2
49.3
35.3
35.3
33.9
29.0
28.9
26.8
26.0
25.1
210
200
190
180
170
160
150
140
130
120
110
100
90
80
70
60
50
40
30
20
f1 (ppm)

Figure S43

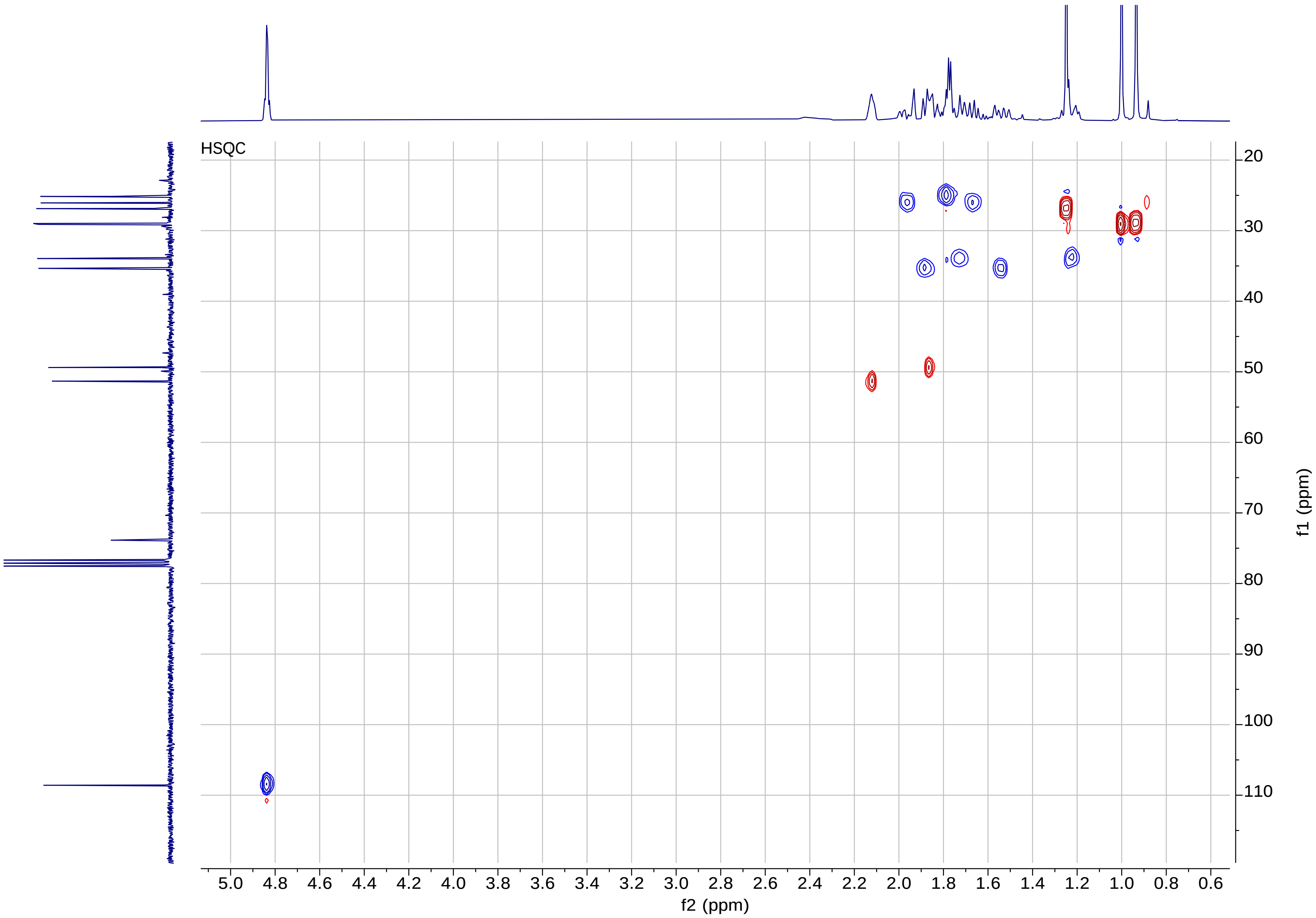
HSQC
5.0
4.8
4.6
4.4
4.2
4.0
3.8
3.6
3.4
3.2
3.0
2.8
2.6
2.4
2.2
2.0
1.8
1.6
1.4
1.2
1.0
0.8
0.6
f2 (ppm)
20
30
40
50
60
70
80
90
100
110
f1 (ppm)

Figure S44

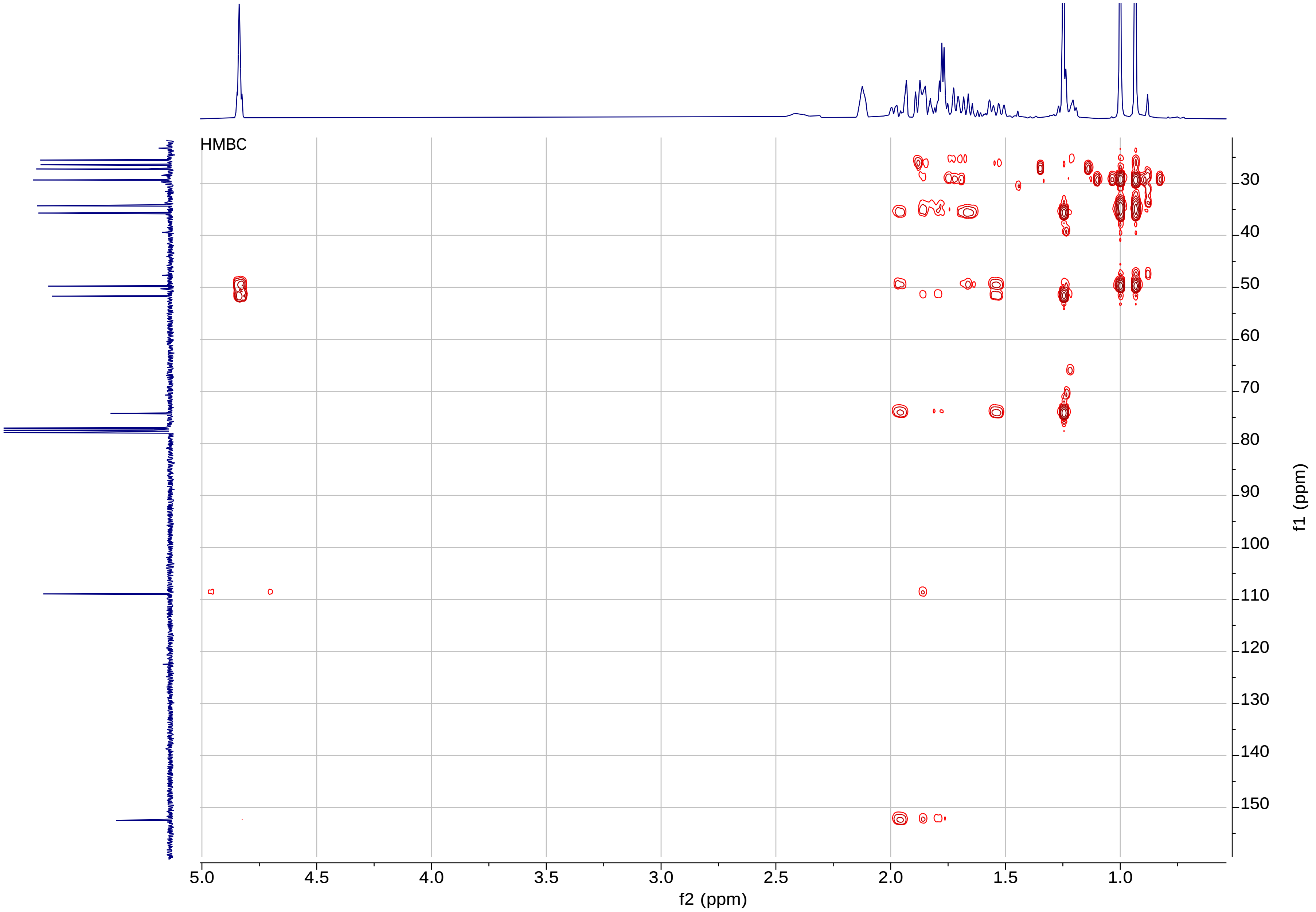
HMBC
5.0
4.5
4.0
3.5
3.0
2.5
2.0
1.5
1.0
f2 (ppm)
30
40
50
60
70
80
90
100
110
120
130
140
150
f1 (ppm)

Figure S45

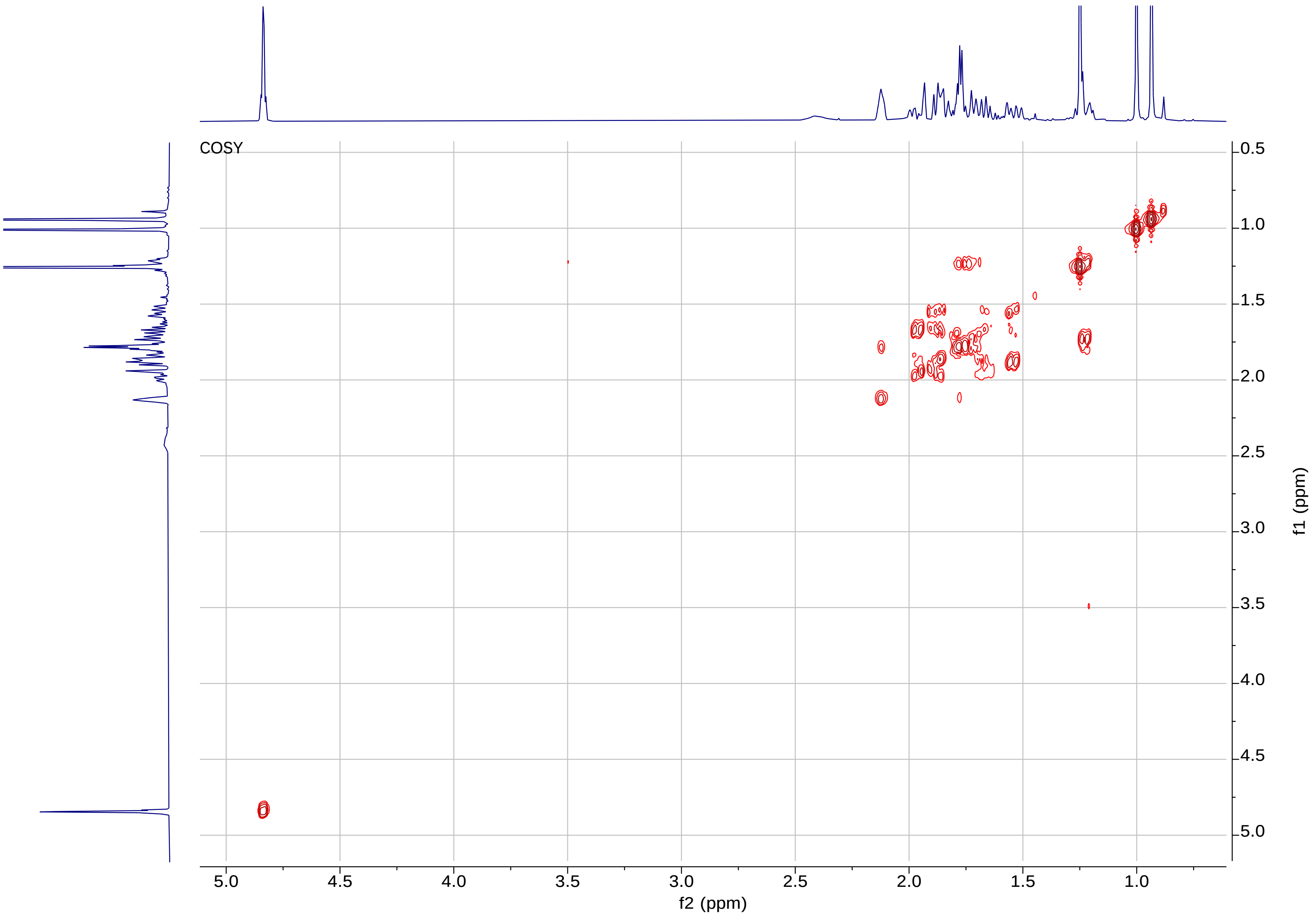

Figure S46

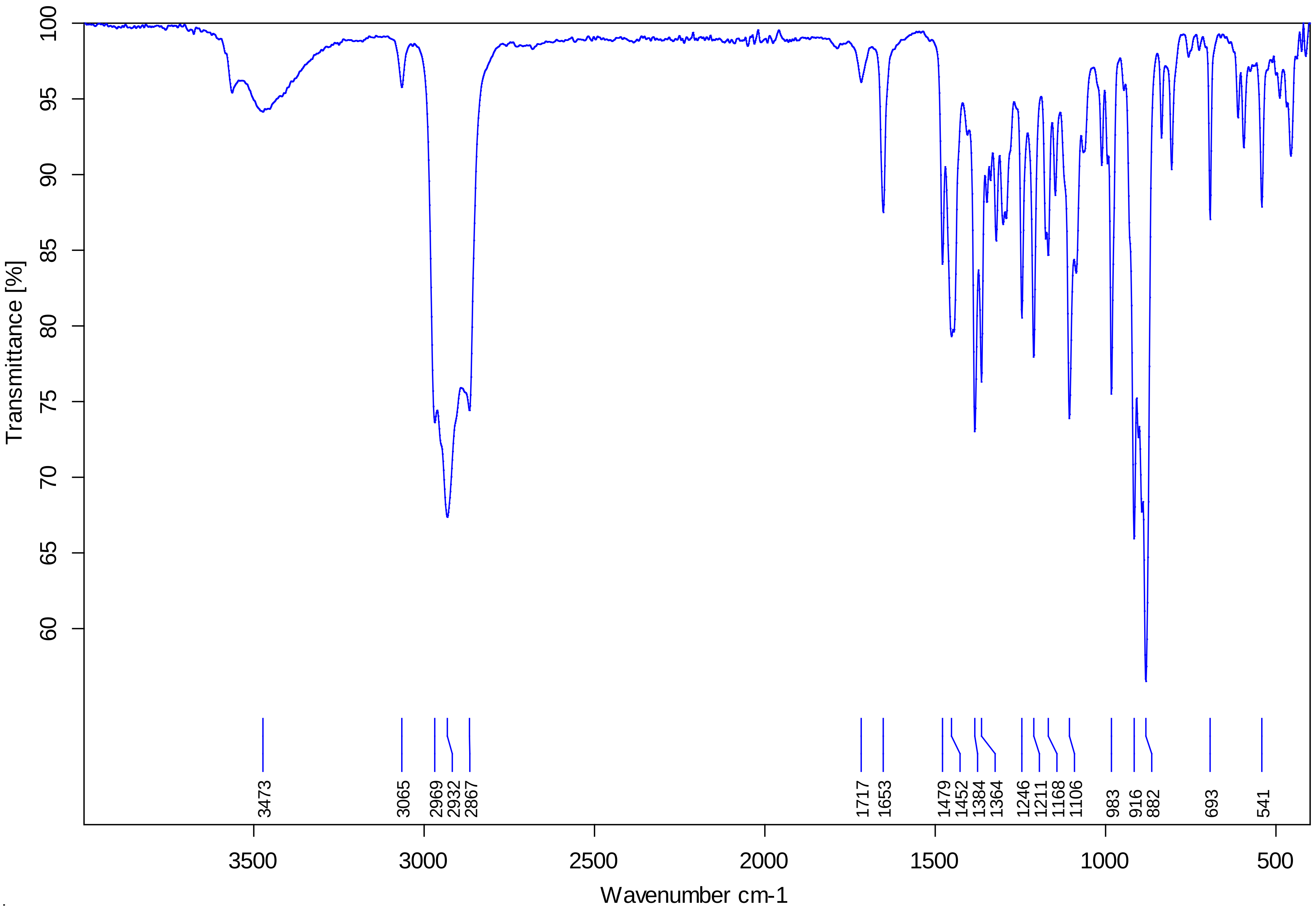
Transmittance [%]
100
95
90
85
80
75
70
65
60
3473
3065
2969
2932
2867
1717
1653
1479
1452
1384
1364
1246
1211
1168
1106
983
916
882
693
541
3500
3000
2500
2000
1500
1000
500
Wavenumber cm-1

Figure S47
